# FDA‐approved drugs featuring macrocycles or medium‐sized rings

**DOI:** 10.1002/ardp.202400890

**Published:** 2025-01-25

**Authors:** Youlong Du, Anas Semghouli, Qian Wang, Haibo Mei, Loránd Kiss, Daniel Baecker, Vadim A. Soloshonok, Jianlin Han

**Affiliations:** ^1^ Jiangsu Co‐Innovation Center of Efficient Processing and Utilization of Forest Resources, College of Chemical Engineering Nanjing Forestry University Nanjing China; ^2^ Institute of Organic Chemistry, Stereochemistry Research Group, HUN‐REN Research Centre for Natural Sciences Budapest Hungary; ^3^ Department of Pharmaceutical and Medicinal Chemistry, Institute of Pharmacy Freie Universität Berlin Berlin Germany; ^4^ Department of Organic Chemistry I, Faculty of Chemistry University of the Basque Country UPV/EHU San Sebastián Spain; ^5^ IKERBASQUE, Basque Foundation for Science Bilbao Spain

**Keywords:** asymmetric synthesis, drug design and development, FDA‐approved drugs, macrocyclic drugs, peptides and proteins

## Abstract

Macrocycles or medium‐sized rings offer diverse functionality and stereochemical complexity in a well‐organized ring structure, allowing them to fulfill various biochemical functions, resulting in high affinity and selectivity for protein targets, while preserving sufficient bioavailability to reach intracellular compartments. These features have made macrocycles attractive candidates in organic synthesis and drug discovery. Since the 20th century, more than three‐score macrocyclic drugs, including radiopharmaceuticals, have been approved by the US Food and Drug Administration (FDA) for treating bacterial and viral infections, cancer, obesity, immunosuppression, inflammatory, and neurological disorders, managing cardiovascular diseases, diabetes, and more. This review presents 17 FDA‐approved macrocyclic drugs during the past 5 years, highlighting their importance and critical role in modern therapeutics, and the innovative synthetic approaches for the construction of these macrocycles.

## Graphical abstract

This review presents 17 macrocyclic drugs approved by the U.S. Food and Drug Administration during the past 5 years, highlighting their importance and critical role in modern therapeutics, and the innovative synthetic approaches for the construction of these macrocycles.

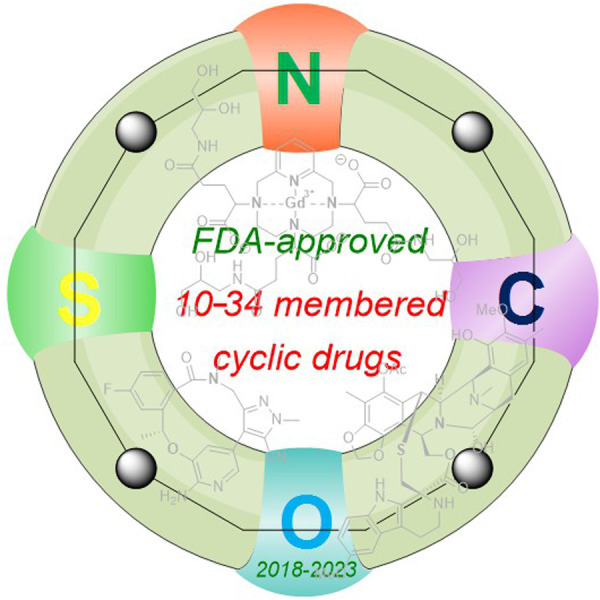

## 1 INTRODUCTION

Macrocycles and medium‐sized rings are an important group of compounds that clearly have great significance in the fields of chemistry, biology, and medicine, offering unique drug‐like profiles that are appealing because of their cyclic structures, flexibility, and preorganization induced by the structural templates.^[^ ^1^, ^2^, ^3^, ^4^, ^5^, ^6^, ^7^, ^8^, ^9^, ^10^ ^]^ By definition, normal‐sized rings consist of five to seven members, while medium‐sized rings cover 8–11 membered cycles and macrocycles are even larger (≥ 12 members).^[^ ^11^, ^12^ ^]^ Such molecules, generally defined as organic compounds containing a ring of at least 12 heavy atoms, have seen growing interest across various scientific fields over the past few decades, with a substantial increase in research and publications.^[^ ^13^, ^14^ ^]^ Macrocycles have become increasingly valuable in drug discovery since the turn of the millennium due to their capability to offer functional diversity, stereochemical complexity, and enhanced pharmacokinetic and pharmacodynamic properties. Compared with ring‐opened analogs, macrocycles often exhibit higher binding affinity and selectivity, leading to significant increase in potency, while maintaining sufficient cell permeability and bioavailability to target intracellular sites after administration.^[^ ^15^, ^16^, ^17^, ^18^, ^19^, ^20^, ^21^, ^22^, ^23^, ^24^, ^25^, ^26^ ^]^ Historically, macrocyclic drugs were originally created from natural sources. Now, however, they are synthetically formed using high‐throughput chemistry and they are getting approved for therapeutic use.^[^ ^5^, ^9^ ^]^

Comparing the ratio of medium‐sized and macrocyclic representatives among these drugs, the smaller rings are clearly underrepresented. One of the reasons for this is the challenge of their synthetic accessibility due to their thermodynamic and kinetic properties.^[^ ^11^ ^]^ On the one hand, the medium‐sized rings are large enough that the ring closure of an open‐chain reactant is accompanied by entropy loss. On the other hand, they are so small that transannular destabilization occurs.^[^ ^27^ ^]^

In this review, we explore the current advancements in drug discovery regarding macrocycles or medium‐sized rings, which were approved by the FDA during the past 5 years. For each compound, we discuss its discovery and biological properties, with a focus on therapeutic indications and the nature of the targets they modulate. Further, we examine the synthetic routes used for their production where applicable.

**Figure 1:**
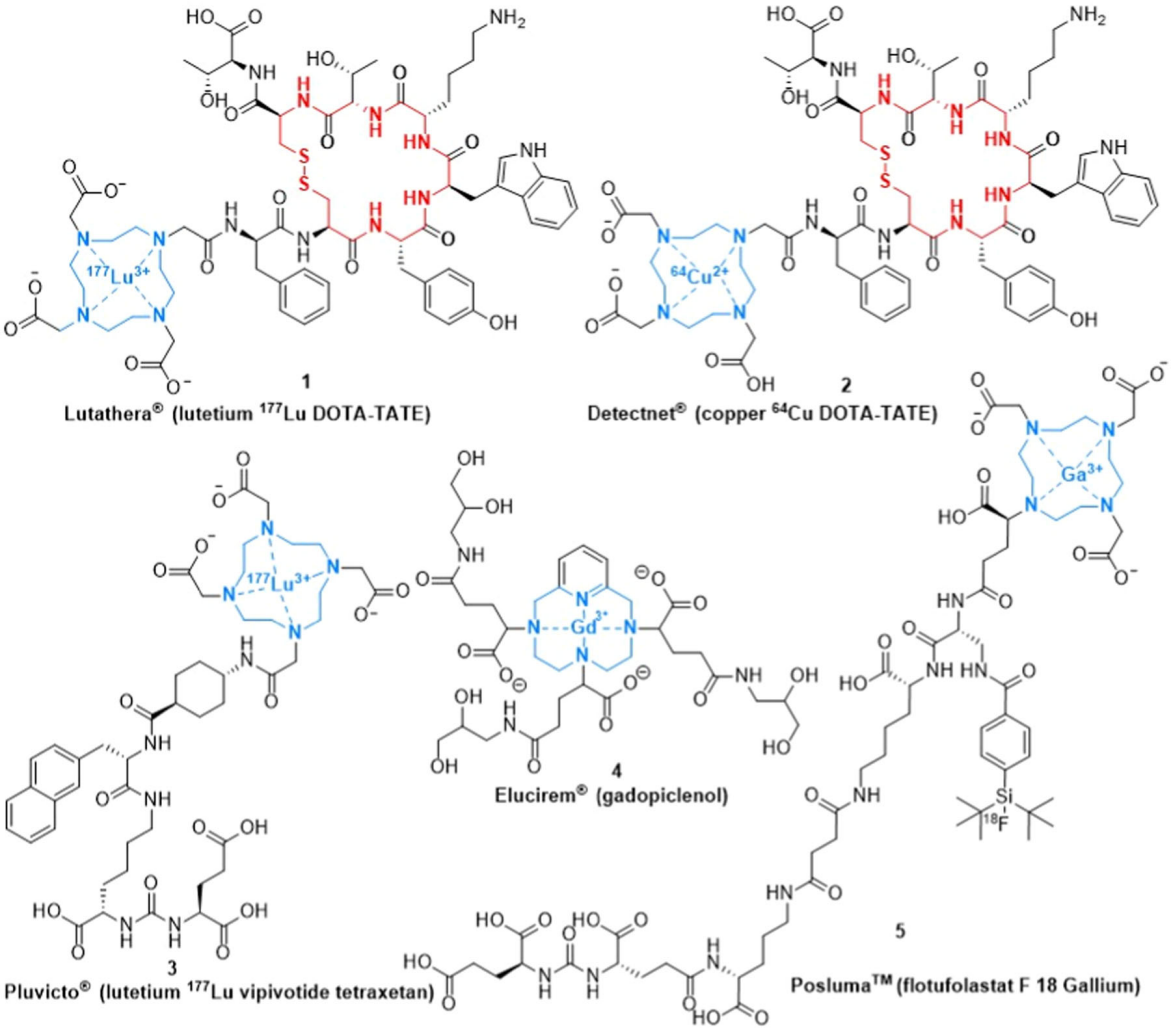
Drugs containing cyclic metal complexes.

**Figure 2:**
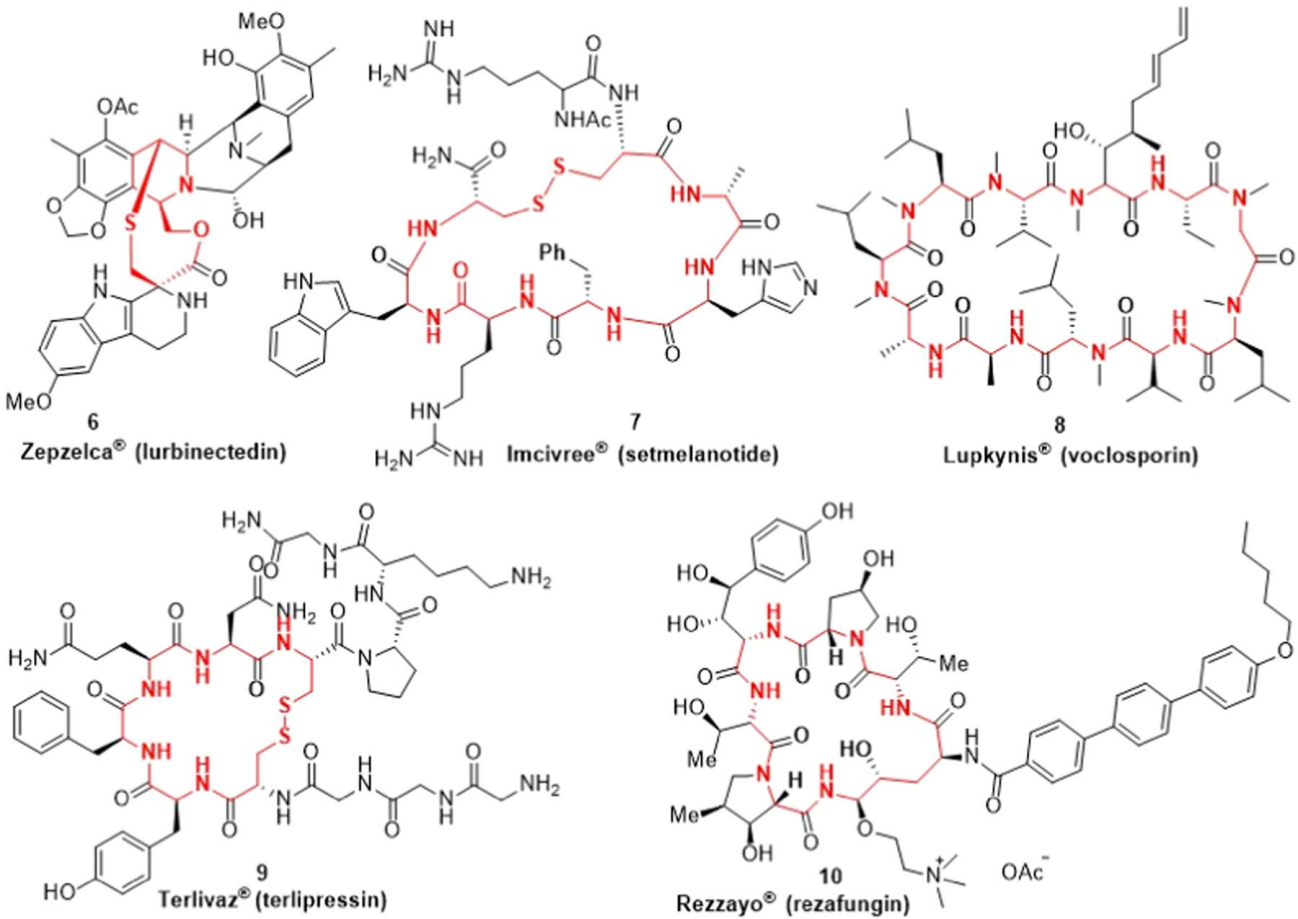
Drugs containing cyclic peptides.

**Figure 3:**
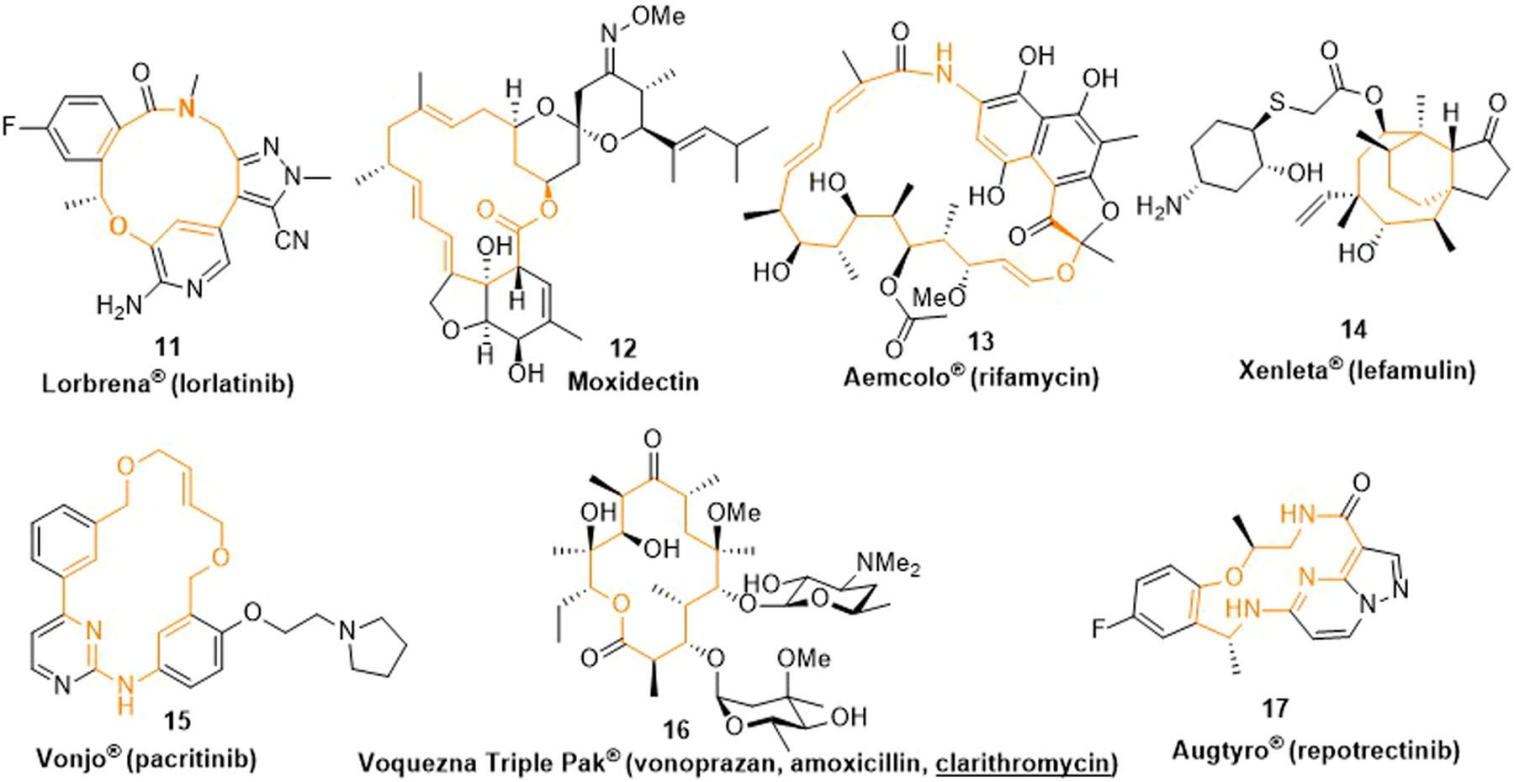
Drugs containing other cyclic units.

As shown in Figures 1, 2, 3, these drugs include five macrocyclic (i.e., ≥ 12‐membered rings) radiopharmaceuticals (Figure 1): ^177^Lu dodecane tetraacetic acid (DOTA)‐TATE (D‐Phe‐Cys‐Tyr‐D‐Trp‐Lys‐Thr‐Cys‐Thr) (Lutathera®) (**1**) for the treatment of gastrointestinal neuroendocrine tumors; ^64^Cu DOTA‐TATE (Detectnet®) (**2**) as a radioactive diagnostic agent used to detect and pinpoint somatostatin receptor‐positive neuroendocrine tumors; Lutetium Lu 177 vipivotide tetraxetan (Pluvicto®) (**3**) employed in the treatment of adult patients with metastatic castration‐resistant prostate cancer; Gadopiclenol (Elucirem®) (**4**) designed to detect and visualize lesions together with magnetic resonance imaging; Flotufolastat F 18 gallium (Posluma®) (**5**) described as a radioactive diagnostic agent for positron emission tomography (PET) in men with prostate cancer.

Also, five cyclic peptide drugs (Figure 2) are presented, which include Lurbinectedin (Zepzelca®) (**6**) used to treat adults with small cell lung cancer (SCLC); Setmelanotide (Imcivree®) (**7**) employed for chronic weight management in patients with obesity due to POMC, PCSK1, or LEPR deficiency; Voclosporin (Lupkynis®) (**8**) indicated for the treatment of active lupus nephritis; Terlipressin (Terlivaz®) (**9**) indicated to improve kidney function in adults suffering from hepatorenal syndrome with rapid reduction in kidney function; Rezafungin (Rezzayo®) (**10**) used for the treatment of candidemia and invasive candidiasis in adults. The drug **6** bears medium‐sized rings, while all of the other peptides (**7–10**) can be counted among the macrocyclic drugs.

In addition, several other macrocyclic and one medium‐sized pharmaceuticals have been described (Figure 3), such as Lorlatinib (Lorbrena®) (**11**) for the initial treatment for people with ALK‐positive metastatic non‐small cell lung cancer (NSCLC); Moxidectin (**12**) indicated for the treatment of onchocerciasis; Rifamycin (Aemcolo®) (**13**) used for the treatment of adult patients with traveler diarrhea caused by noninvasive strains of *Escherichia coli*; Lefamulin (Xenleta®) (**14**) designed for the treatment of community‐acquired bacterial pneumonia; Pacritinib (Vonjo®) (**15**) as a kinase inhibitor indicated for the treatment of adults with myelofibrosis and thrombocytopenia; Clarithromycin (Voquezna®) (**16**) for the treatment of *Helicobacter pylori* infections in adults and to manage erosive esophagitis; Repotrectinib (Augtyro®) (**17**) designed for the treatment of adult patients with locally advanced or metastatic ROS1‐positive NSCLC. Among them, compounds **11–13** and **15–17** represent macrocycles, while drug **14** has a medium‐sized ring.

## 2 MACROCYCLIC RADIOPHARMACEUTICALS

### 2.1 ^177^Lu DOTA‐TATE (Lutathera®)

¹⁷⁷Lu dotatate (**1**), also known as Lutetium oxodotreotide (brand name Lutathera®), is a chelated complex consisting of a radioisotope of the element lutetium combined with DOTA‐(Tyr3)‐octreotate (DOTA‐TATE), a peptide that is effective in binding to specific receptors in particular tumors.^[^ ^28^, ^29^ ^]^ Lutathera®, approved in January 2018 by the FDA, was launched by Advanced Accelerator Applications.^[^ ^30^ ^]^ It was indicated for adults to treat gastrointestinal neuroendocrine tumors (GEP‐NETs), which are unresectable or metastatic, progressive, well‐differentiated (Grades I and II), and positive for somatostatin receptors. It is a radiopharmaceutical medicine with the lutetium radioisotope delivering localized radiation to tumor cells, minimizing damage to surrounding healthy tissue. Due to the radioactive nature of ^177^Lu, special precautions are necessary during and after its administration to minimize environmental contamination from excreted materials and to reduce radiation exposure to healthcare personnel and the public caused by gamma photon emissions. As a result, Lutathera® needs to be given in a hospital that has nuclear medicine facilities only by professionals, who are authorized to handle radiopharmaceuticals.^[^ ^31^ ^]^

**Scheme 1:**
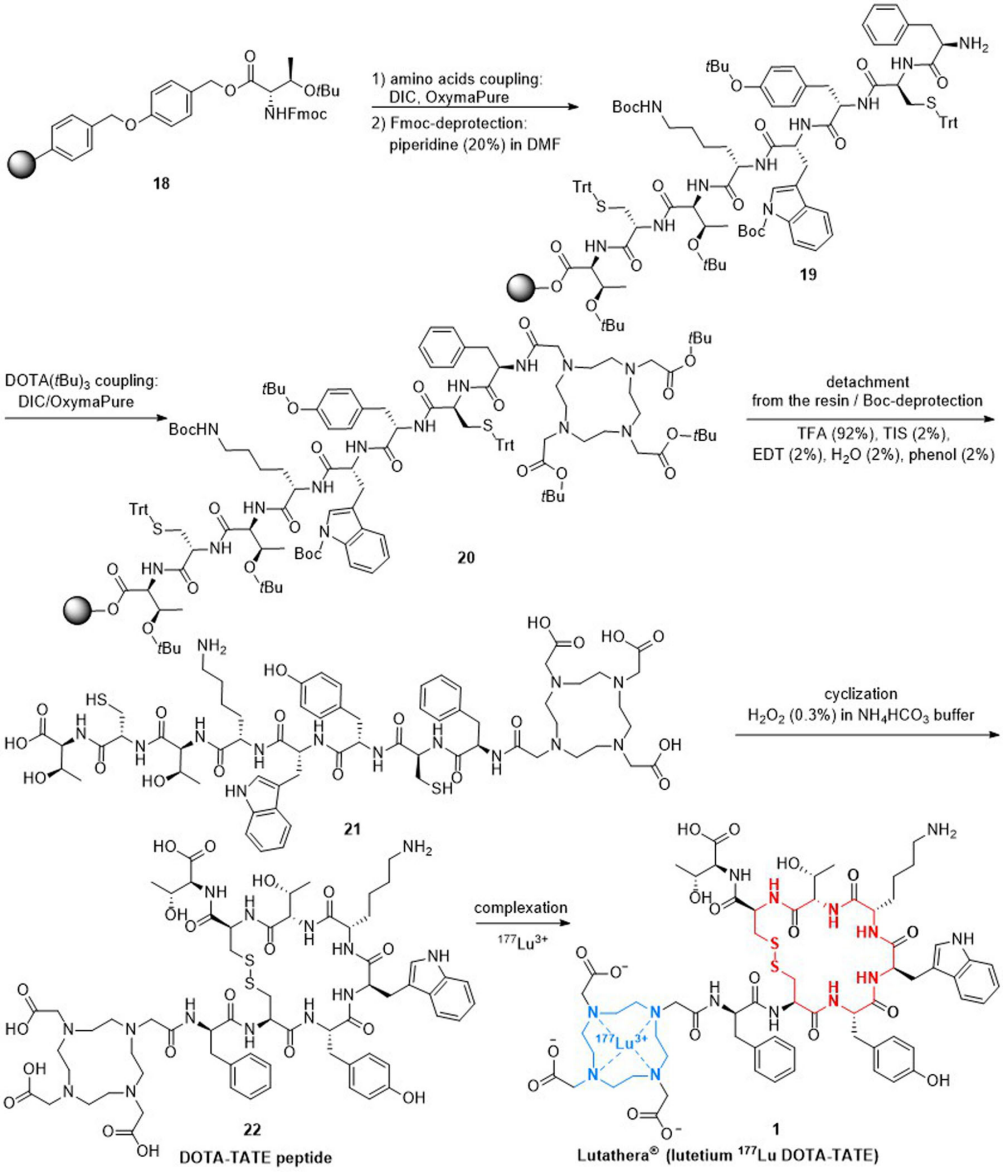
Synthesis of DOTA‐TATE peptide and ^177^Lu DOTA‐TATE (**1**).

The synthetic preparation of DOTA‐TATE **22**, as illustrated in Scheme 1, involved the use of solid‐phase peptide chemistry.^[^ ^32^, ^33^, ^34^, ^35^ ^]^ First, in the coupling reaction, amino acids were activated using *N*,*N*‐diisopropylcarbodiimide (DIC) with OxymaPure (ethyl cyano(hydroxyimino)acetate). After amino acid coupling, Fmoc‐deprotection of the previous amino acid residing on the resin was performed using a solution of piperidine (20%) in *N*,*N*‐dimethylformamide (DMF). It was followed by a DOTA coupling step using a double amount of OxymaPure to minimize the risk of racemization of amino acids in the Fmoc‐deprotection step^[^ ^32^, ^33^ ^]^ resulting in the compound **20**. Next, the peptide was cleaved from the resin, followed by Boc‐deprotection and cleavage of the *tert*‐butyl ester carried out at room temperature in the presence of a mixture of trifluoroacetic acid (TFA) with additions of 2% by volume each of triisopropylsilane (TIS), phenol, water, and 1,2‐ethanedithiol (EDT) yielding the compound **21**. The cyclization step and the formation of the disulfide bridge were carried out by oxidation of cysteines with H_2_O_2_ (0.3%) dissolved in ammonium bicarbonate buffer (NH_4_HCO_3_) resulting in DOTA‐TATE **22**. Finally, the complexation of the DOTA‐TATE peptide with ^177^Lu radioisotopes closed the synthesis process for ^177^Lu DOTA‐TATE (**1**).

### 2.2 ^64^Cu DOTA‐TATE (Detectnet®)

^64^Cu DOTA‐TATE (**2**, brand name Detectnet®) is a macrocyclic compound composed of radionuclide ^64^Cu chelated by DOTA‐TATE.^[^ ^36^, ^37^ ^]^ Detectnet® approved in September 2020 by the FDA was launched by RadioMedix, Inc.^[^ ^38^ ^]^ It is a radioactive diagnostic agent designed for the use with PET imaging to detect and pinpoint somatostatin receptor‐positive neuroendocrine tumors (NETs) in adults,^[^ ^39^, ^40^ ^]^^64^Cu is one of the significant copper radioisotopes. It is particularly suitable for clinical applications because it has an intermediate half‐life of 12.7 h. It decays through multiple pathways, which include electron capture, beta emission, and positron emission. In addition, the low positron energy of Cu and the absence of gamma emission allow to produce images of high quality and high resolution.^[^ ^41^ ^]^^64^Cu DOTA‐TATE is considered to be superior to ^68^Ga DOTA‐TOC, which was approved in 2019. This superiority is attributed to the radionuclide properties of ^68^Ga with a shorter half‐life of 1.13 h and the production of lower‐resolution image analysis due to the higher positron energy of Ga.^[^ ^42^, ^43^ ^]^ In general, Detectnet® (**2**) is used for diagnosing and imaging somatostatin receptor‐positive NETs, helping physicians to locate tumors accurately in the body, while Lutathera® (**1**) is used to directly target and treat gastrointestinal neuroendocrine tumors (GEP‐NETs).

**Scheme 2:**
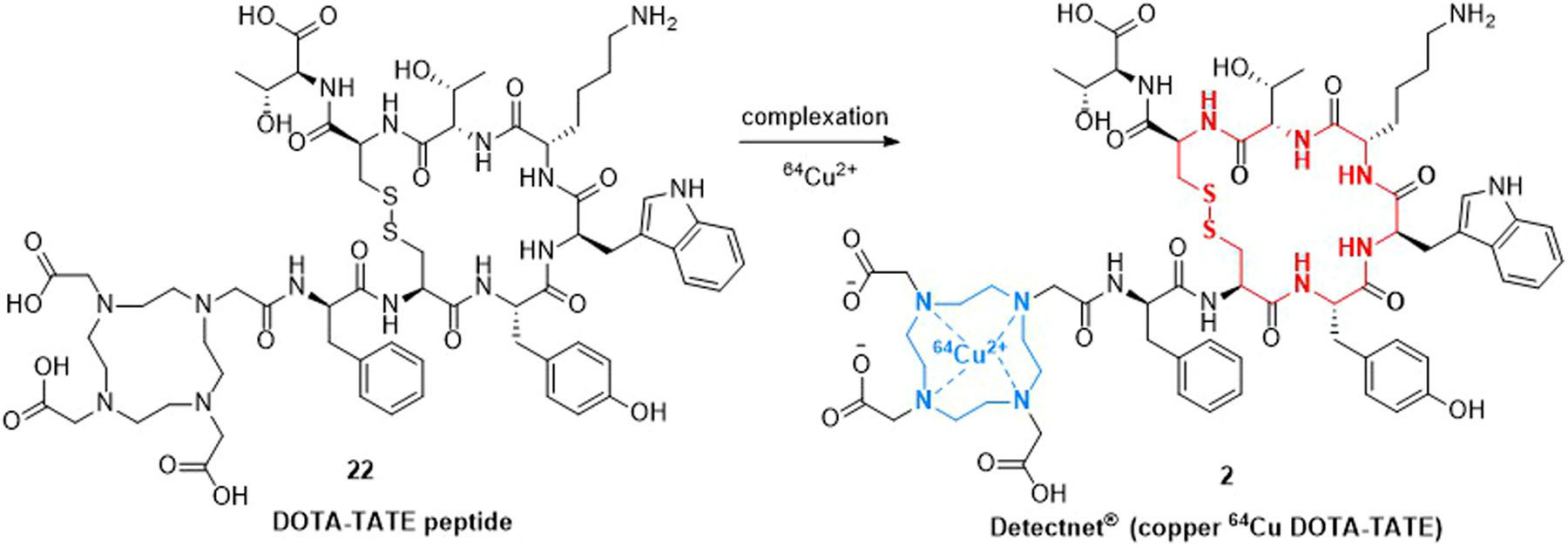
^64^Cu DOTA‐TATE (**2**).

The synthetic preparation of ^64^Cu DOTA‐TATE (**2**), as shown in Scheme 2, involves the complexation of the DOTA‐TATE peptide **22** with ^64^Cu radioisotopes.^[^ ^44^ ^]^

### 2.3 Lutetium Lu 177 vipivotide tetraxetan (Pluvicto®)

**Scheme 3:**
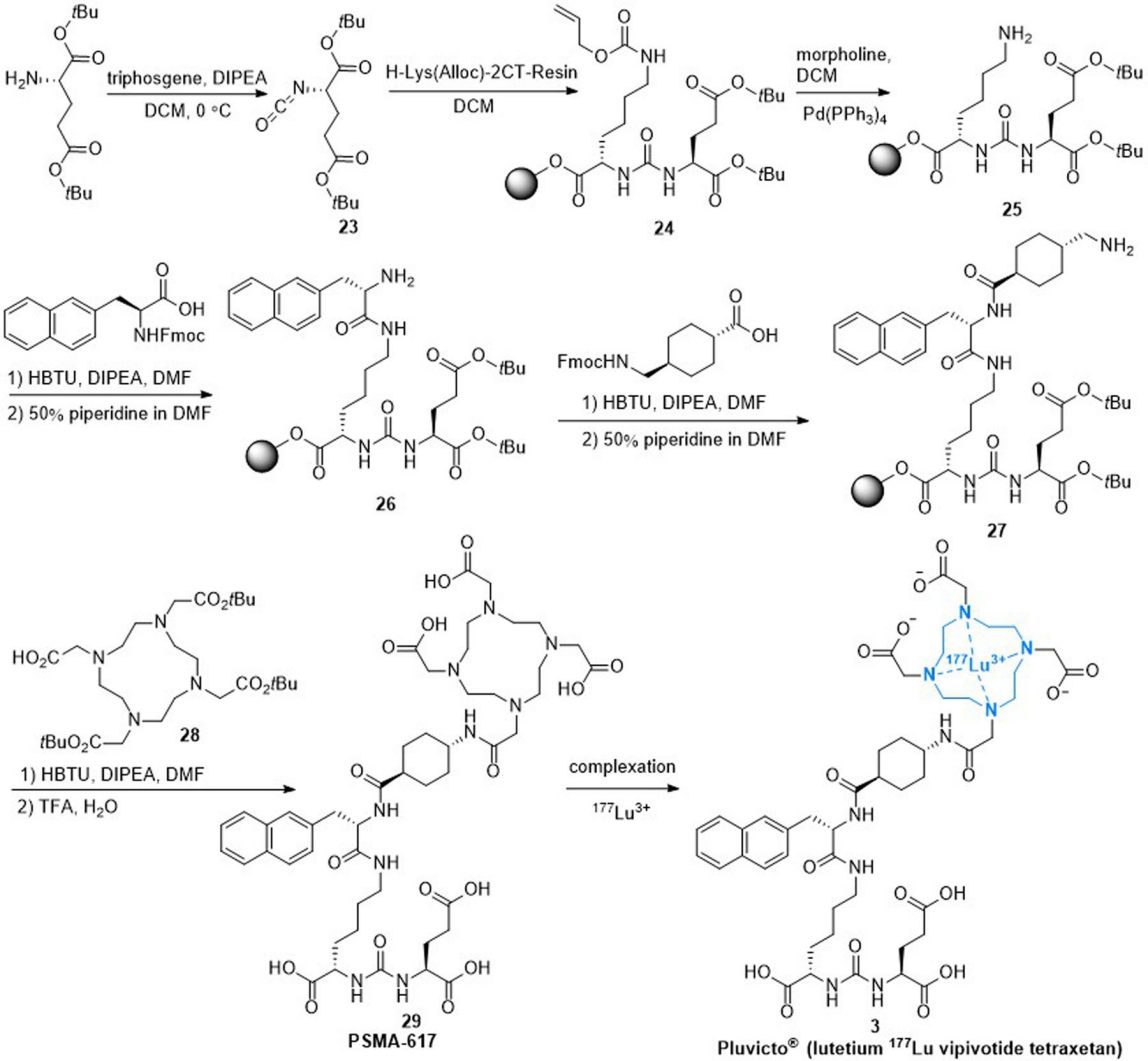
Synthesis of PSMA‐617 and Lutetium Lu 177 vipivotide tetraxetan (**3**).

Lutetium Lu 177 vipivotide tetraxetan (**3**, brand name Pluvicto®) is a macrocycle composed of a prostate‐specific membrane antigen (PSMA) namely complex PSMA‐617 with the beta‐emitting radioisotope lutetium ^177^Lu.^[^ ^45^ ^]^ Pluvicto® ([^177^Lu]Lu‐PSMA‐617) approved in March 2022 by FDA was launched by Advanced Accelerator Applications USA, Inc., a Novartis company.^[^ ^46^ ^]^ It was employed in the treatment of adult patients with metastatic castration‐resistant prostate cancer (mCRPC) who have a high level of PSMA and one or more metastatic lesions. This is the initial radioligand therapy that has been approved by the FDA for men who are eligible and have PSMA‐positive mCRPC. Lutetium Lu 177 vipivotide tetraxetan (**3**) is a radioligand that strongly binds to PSMA, making it ideal for treating prostate cancer through targeted radiation, which results in DNA damage and cell death.^[^ ^47^, ^48^ ^]^ The synthesis process for PSMA‐617 (**29**) (Scheme 3) involves the use of solid‐phase peptide chemistry. First, the amino group of l‐glutamate, protected by two *tert*‐butyl moieties, was converted to isocyanate **23** using triphosgene. Next, the reaction of isocyanate **23** with resin‐immobilized lysine protected by the ε‐allyloxycarbonyl group formed the urea intermediate **24**. The allyloxy‐protecting group was removed with *tetrakis*(triphenylphosphine)palladium(0) (Pd(PPh_3_)_4_) and the reaction with morpholine yielded the intermediate **25**. Then the intermediate **25** was condensed with Fmoc‐3‐(2‐naphthyl)‐l‐alanine (Fmoc‐2‐Nal‐OH) using *N*,*N*,*N*′,*N*′‐tetramethyl‐*O*‐(1*H*‐benzotriazol‐1‐yl)uronium hexafluorophosphate (HBTU) and then treated with piperidine produced naphthyl‐based pseudopeptide **26**. The free amino group of **26** was condensed with *trans*‐4‐(Fmoc‐aminomethyl)cyclohexanecarboxylic acid, followed by Fmoc‐deprotection to give the intermediate **27**. Finally, the free aminomethyl group of **27** was coupled with tri*‐tert*‐butyl 1,4,7,10‐tetraazacyclododecane‐1,4,7,10‐tetraacetate (DOTA‐tris(*t*Bu)ester) **28** resulting in PSMA‐617 **29**.^[^ ^49^, ^50^ ^]^ Complexation of the PSMA‐617 **29** with ^177^Lu radioisotopes affords the target product Lutetium Lu 177 vipivotide tetraxetan (**3**).

### 2.4 Gadopiclenol (Elucirem®)

**Scheme 4:**
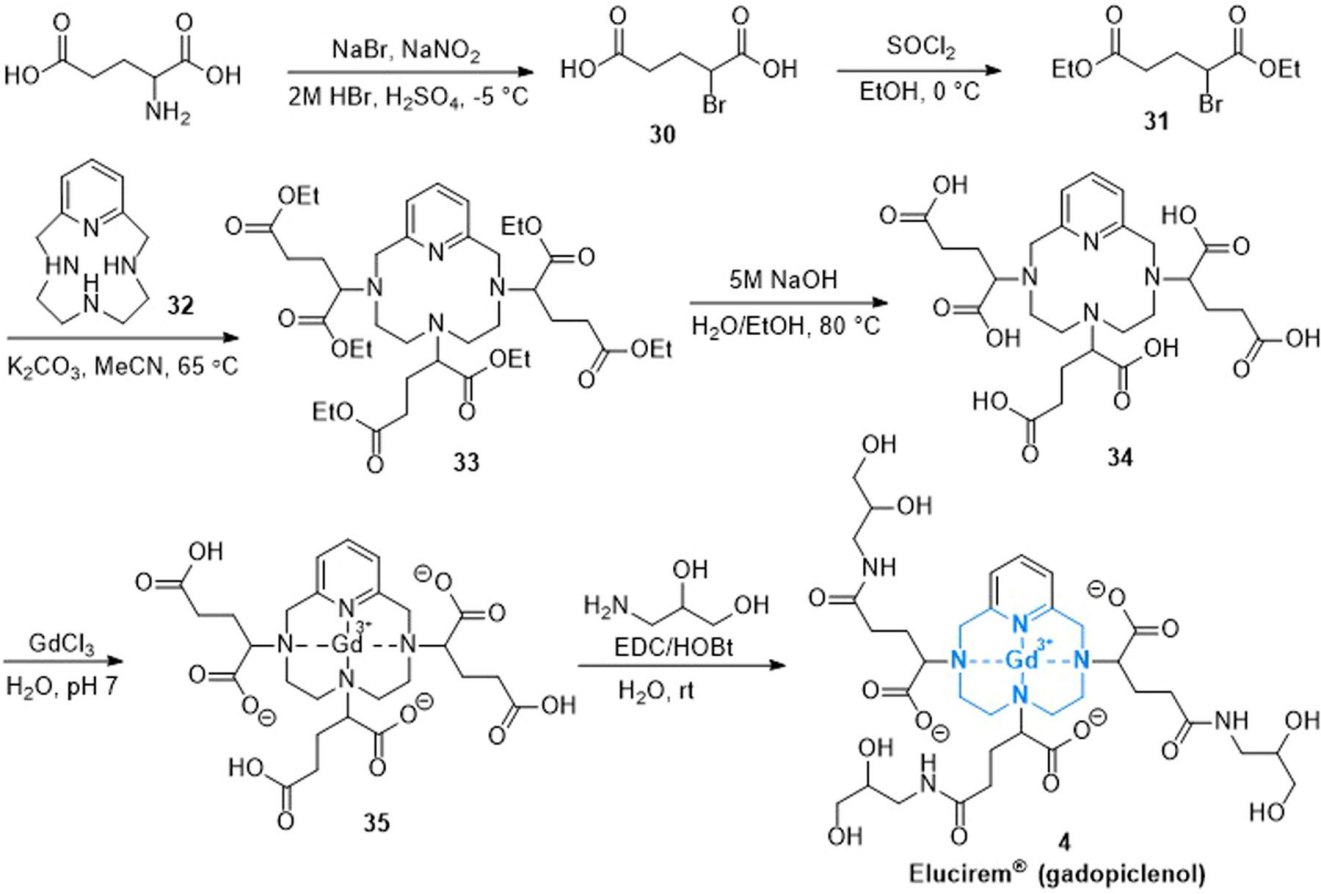
Synthesis of gadopiclenol (**4**).

Gadopiclenol (**4**, brand name Elucirem®) is a macrocyclic gadolinium complex with a pyridine‐containing triaza (PCTA) and three ionizable (2,3‐dihydroxypropyl)amino)‐5‐oxopentanoic acid pendants.^[^ ^51^ ^]^ Gadopiclenol has been approved in September 2022 by the FDA. It was launched by Bracco Imaging SpA with high relaxivity properties, which was designed to detect and visualize lesions, together with magnetic resonance imaging (MRI), with abnormal vascularity in the central nervous system and the body.^[^ ^52^ ^]^ It is an extracellular fluid (ECF) contrast agent that quickly leaves the bloodstream and accumulates in extracellular spaces. Tumors, with abnormal vascularity, retain higher concentrations of the contrast agent than healthy tissues, enhancing visualization.^[^ ^53^ ^]^ Gadopiclenol is administered intravenously in single‐dose prefilled syringes. Its diagnostic efficacy and safety have been evaluated in two Phase III clinical studies by the pharmaceutical company Guerbet producing the complex.^[^ ^54^ ^]^ The synthetic preparation as illustrated in Scheme 4 began with racemic glutamic acid. The amino group was transformed into the diazo derivative and then it was reacted with hydrobromic acid to produce the derivative **30**. This compound **30** was converted to diethyl ester **31** using thionyl chloride in ethanol. Next, a nucleophilic substitution of compound **31** with 3,6,9‐triaza‐1(2,6)‐pyridinacyclodecaphane (**32**) afforded the intermediate **33**, which was hydrolyzed with NaOH to yield hexa‐acid **34**. The following complexation with gadolinium (III) chloride results in the intermediate **35**. Finally, this intermediate was condensed with isoserinol under 1‐(3‐dimethylaminopropyl)‐3‐ethylcarbodiimide (EDC)/1‐hydroxybenzotriazole (HOBt) coupling conditions to produce isomeric gadopiclenol (**4**).^[^ ^55^ ^]^

### 2.5 Flotufolastat F 18 (Posluma®)

**Scheme 5:**
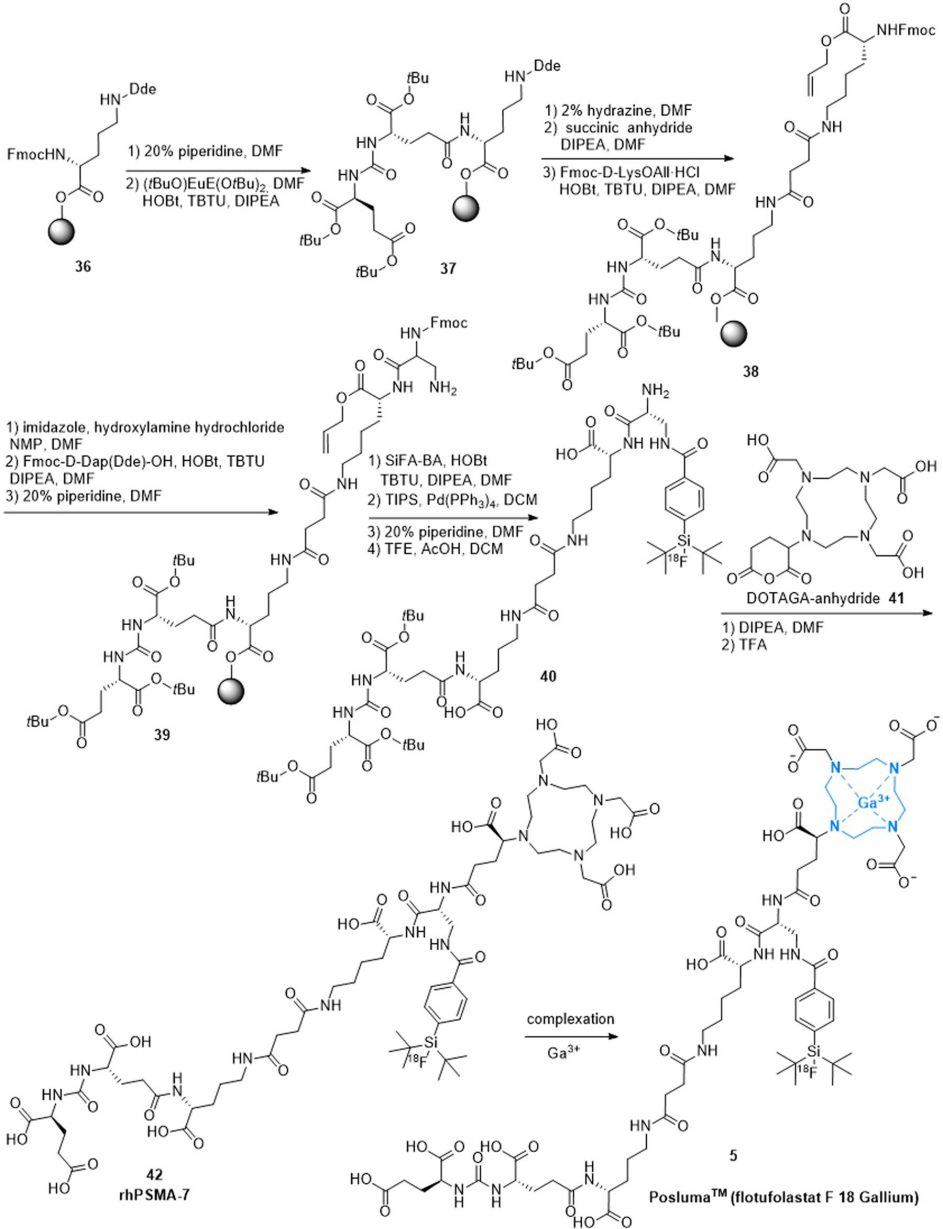
Synthesis of rhPSMA‐7 and flotufolastat F 18 (**5**).

Flotufolastat F 18 (**5**, brand name Posluma®) is a radioactive diagnostic agent composed of a PSMA‐binding ligand. It consists of a DOTAGA complex with nonradioactive Ga^3+^ and a radioactive ^18^F atom that is covalently attached to silicon.^[^ ^56^ ^]^ Posluma® (^18^F‐rhPSMA‐7.3), approved in May 2023 by the FDA, was developed by Blue Earth Diagnostics, a subsidiary of Bracco Imaging, for prostate cancer imaging (Milan, Italy). It was described as a radioactive diagnostic agent for PET in men with prostate cancer. It is used to detect PSMA‐positive lesions in patients who either have suspected metastasis and are candidates for initial definitive therapy or have a suspected recurrence indicated by elevated serum prostate‐specific antigen (PSA) levels.^[^ ^56^, ^57^ ^]^ Interestingly, it is the first diagnostic agent approved with proprietary radiohybrid (rh) technology, which features two binding sites for radionuclides.^[^ ^58^ ^]^ The radiohybrid PSMA‐targeted ligand [^18^F]‐rhPSMA‐7 is a mixture of four stereoisomers ([^18^F]‐rhPSMA‐7.1, ‐7.2, ‐7.3 and ‐7.4) and after a preclinical isomer selection process, [^18^F]‐rhPSMA‐7.3 has entered drug formulation.^[^ ^59^ ^]^ The solid‐phase peptide synthesis process was used in the fabrication of rhPSMA‐7 (Scheme 5). First, Fmoc‐deprotected **36** with 20% piperidine in DMF and (*t*BuO)EuE(O*t*Bu)_2_ was conjugated with HOBt, *O*‐(benzotriazol‐1‐yl)‐*N,N,N’,N’*‐tetramethyluronium tetrafluoroborate (TBTU), and *N,N*‐diisopropylethylamine (DIPEA) in DMF for 4.5 h. After cleavage of the Dde group with a mixture of 2% hydrazine in DMF, a solution of succinic anhydride and DIPEA in DMF was added followed by a reaction for 2.5 h. Conjugation of Fmoc‐d‐Lys‐OAll∙HCl was achieved by adding a mixture of HOBt, TBTU, and DIPEA in DMF delivering the intermediate **38**. After cleavage of the Fmoc group with 20% piperidine in DMF, the free amine was conjugated to Fmoc‐d‐Dap(Dde)‐OH after preactivation of the amino acid in a mixture of HOBt, TBTU, and DIPEA in DMF. Following Dde‐deprotection, treatment with imidazole and hydroxylamine hydrochloride dissolved in a mixture of *N*‐methylpyrrolidone (NMP) and DMF yielded the scaffold **39**. SiFA‐BA was reacted with the free amine **39** in the presence of HOBt, TBTU, and DIPEA as activation reagents in DMF. The allyl‐protecting group was removed by the addition of triisopropylsilylacetylene (TIPS) and Pd(PPh_3_)_4_ dissolved in dichloromethane (DCM). After Fmoc‐deprotection with piperidine, the peptide was cleaved from the resin under preservation of the acid‐labile protecting groups by using a mixture of trifluoroethanol (TFE) and AcOH in DCM affording compound **40**. Finally, condensation of DOTAGA‐anhydride **41** was achieved with piperidine in DMF. After cleavage of the *tert‐*butyl ester functions of the EuE moiety with TFA to form carboxylic acid gave rhPSMA‐7 (**42**).^[^ ^58^, ^60^ ^]^ Complexation with gallium(III) yielded flotufolastat F 18 (**5**).

## 3 CYCLIC PEPTIDE DRUGS

### 3.1 Lurbinectedin (Zepzelca®)

Lurbinectedin (**6**, brand name Zepzelca®) was developed by PharmaMar and received FDA approval in June 2020 for the treatment of metastatic SCLC.^[^ ^61^ ^]^ It exerts its effect by covalently binding to select DNA sequences in the minor groove, inducing double‐stranded DNA breaks that lead to apoptotic cell death.^[^ ^62^ ^]^ Additionally, Lurbinectedin is believed to stimulate anticancer immunity by inducing immunogenic cell death (ICD), which is associated with a significant reduction in tumor‐associated macrophages (TAMs).^[^ ^63^, ^64^ ^]^ Lurbinectedin (**6**) belongs to the compounds bearing medium‐sized rings, as it features an eight‐membered ring and a ten‐membered sulfur‐containing heterocyclic ring. Besides, it has seven chiral centers. Its complex polycyclic structure is reminiscent of trabectedin (ecteinascidine 743), which was first synthesized in the mid‐1990s at Harvard.^[^ ^65^, ^66^ ^]^ As an oncogenic transcription inhibitor, lurbinectedin is a modified version of ecteinascidin, featuring a tetrahydro β‐carboline substituent ^[^ ^67^ ^]^ integrated into its tetrahydroisoquinoline backbone, and also containing a cysteine ester group. The cysteine ester group possesses unique biological functions, as it is capable of forming covalent bonds with crucial functional groups in proteins. The incorporation of this group may facilitate lurbinectedin in more efficiently recognizing and binding to specific biological targets, including DNA or proteins, thereby bolstering its targeting capabilities as an anticancer drug.^[^ ^68^ ^]^

**Scheme 6:**
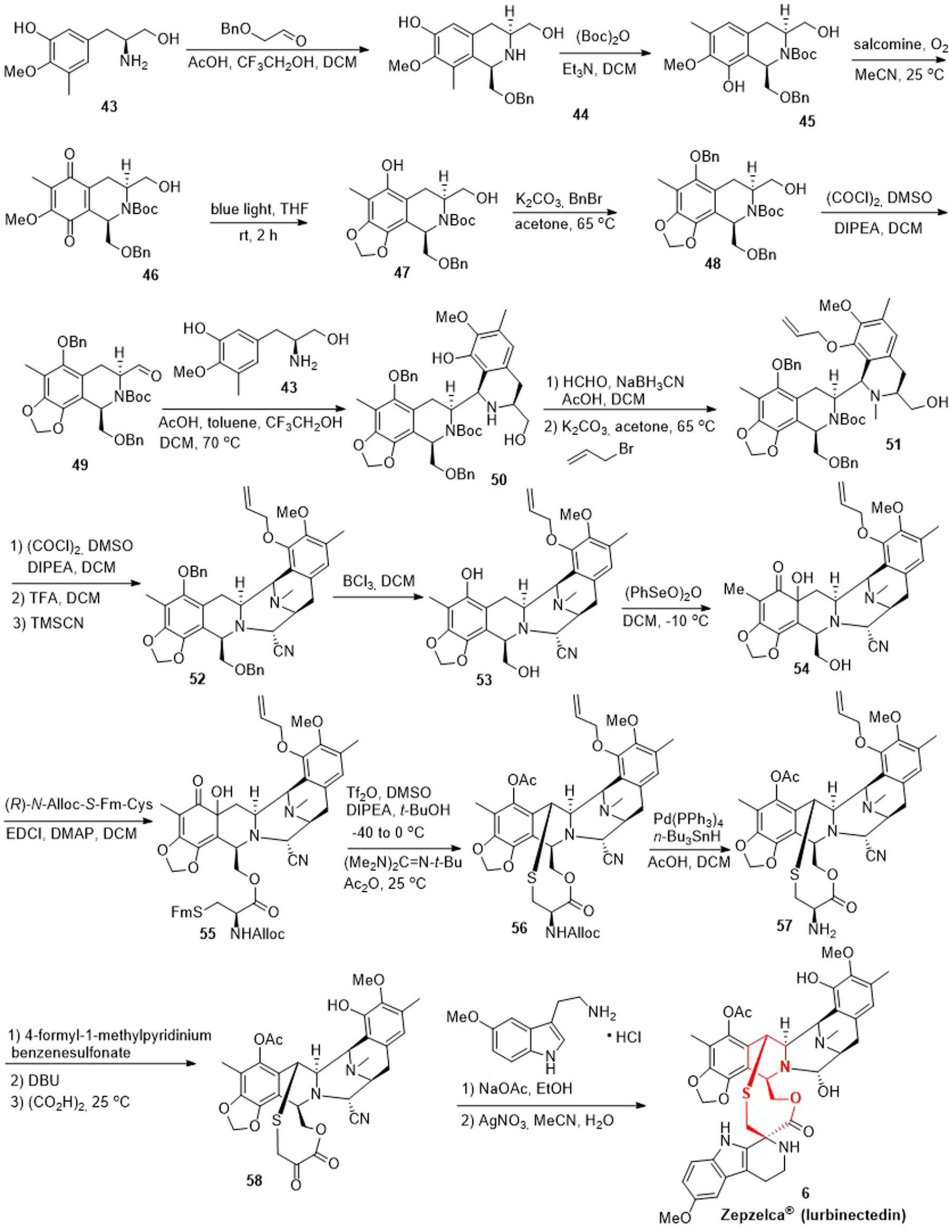
Synthesis of lurbinectedin (**6**).

There have been several methods for the synthesis of lurbinectedin (**6**),^[^ ^69^, ^70^ ^]^ in particular, Ma and co‐authors reported a scalable strategy for the preparation with (*S*)‐tyrosine derivative **43** as starting material (Scheme 6).^[^ ^66^ ^]^ Pictet‐Spengler reaction of (*S*)‐5‐(2‐amino‐3‐hydroxypropyl)‐2‐methoxy‐3‐methylphenol (**43**) with 2‐(benzyloxy)acetaldehyde afforded tetrahydroisoquinoline **44**, which was converted into Boc‐protected tetrahydroisoquinoline **45** via treatment with (Boc)_2_O in DCM. Oxidation of tetrahydroisoquinoline **45** catalyzed by salcomine (*N*,*N*’‐bis(salicylidene)ethylenediamino cobalt (II)) in the presence of oxygen‐delivered quinone **46**. Then, the construction of benzo^[^ ^1^, ^3^ ^]^dioxole via a light‐promoted remote C–H bond activation of quinone **46** resulting in the intermediate **47**, which underwent protection with the benzyl group to generate the intermediate **48**. The alcohol intermediate **48** underwent Swern oxidation successfully gave the aldehyde **49**, which was subjected to the second Pictet‐Spengler reaction with (*S*)‐5‐(2‐amino‐3‐hydroxypropyl)‐2‐methoxy‐3‐methylphenol (**43**) to construct another tetrahydroisoquinoline unit. It should be mentioned that the reaction provided intermediate **50** as the major isomer. Subsequently, reductive amination of amine **50** with formaldehyde in the presence of NaBH_3_CN introduced a methyl group, which then reacted with allyl bromide to give the *O‐allyl*‐protected intermediate **51**. The intermediate **51** underwent Swern oxidation, treatment with TFA, intramolecular cyclization, and Strecker reaction resulting in the polycyclic intermediate **52**. Deprotection of two benzyl groups via treatment with boron trichloride afforded the key alcohol intermediate **53**. Then, oxidation of phenol intermediate **53** by benzeneseleninic anhydride in DCM furnished the ketone intermediate **54** as well as introduction of a hydroxyl group at α‐position. Subsequently, the condensation reaction of the intermediate **54** with (*R*)‐*N*‐Alloc‐*S*‐Fm‐Cys by using 1‐ethyl‐3‐(3‐dimethylaminopropyl)carbodiimide (EDCI) and 4‐dimethylaminopyridine (DMAP) afforded the amino ester **55**. Then, the macrocyclic unit **56** was constructed based on Corey's one‐pot procedure, which underwent deprotection of allyl and alloc groups in the presence of Pd(PPh_3_)_4_ and *N*‐Bu_3_SnH generating amine intermediate **57**. Amine intermediate **57** was converted to ketone **58**, which was subjected to the third Pictet–Spengler reaction with 2‐(5‐methoxy‐1*H*‐indol‐3‐yl)ethanamine hydrochloride salt successfully constructing the tetrahydro‐β‐carboline moiety. Finally, Ag‐promoted hydrolysis of the cyano into hydroxyl group led to the target lurbinectedin (**6**).

### 3.2 Setmelanotide (Imcivree®)

Setmelanotide (**7**, brand name Imcivree®), developed by Rhythm Pharmaceuticals, is a melanocortin‐4 (MC4) receptor agonist. Setmelanotide was approved for treating rare genetic obesity disorders caused by deficiencies in proopiomelanocortin (POMC), proprotein convertase subtilisin/kexin type 1 (PCSK1), or leptin receptor (LEPR) deficiency. These deficiencies lead to hyperphagia, severe childhood obesity, and potential endocrine disorders.^[^ ^71^ ^]^ Setmelanotide (**7**) contains a 23‐membered *N*,*S*‐heterocyclic macrocyclic ring molecule featuring eight chiral centers, which is constructed from a diverse array of natural amino acids, encompassing tryptophan, histidine, cysteine, phenylalanine, and arginine. This molecule adeptly mimics the body's endogenous ligand for the MC4 receptor, α‐MSH, thereby revitalizing the activity of the MC4 receptor pathway. This revitalization leads to a reduction in hunger sensations and facilitates weight loss through a dual mechanism of decreased caloric intake and enhanced energy expenditure.^[^ ^72^ ^]^ The efficacy of setmelanotide may stem from the fact that its disulfide bond constrains its structural conformation, enhancing its affinity for the target receptor and simultaneously hindering its degradation by the proteasome.^[^ ^73^ ^]^ Setmelanotide was approved by the FDA in December 2020 for chronic weight management in patients with obesity due to POMC, PCSK1, or LEPR deficiency, and in June 2022 for the Bardet‐Biedl syndrome. Setmelanotide is the first available treatment for chronic weight management in these conditions, without side effects like blood pressure elevation or heart rate acceleration seen with earlier MC4R agonists.^[^ ^74^, ^75^, ^76^ ^]^

**Scheme 7:**
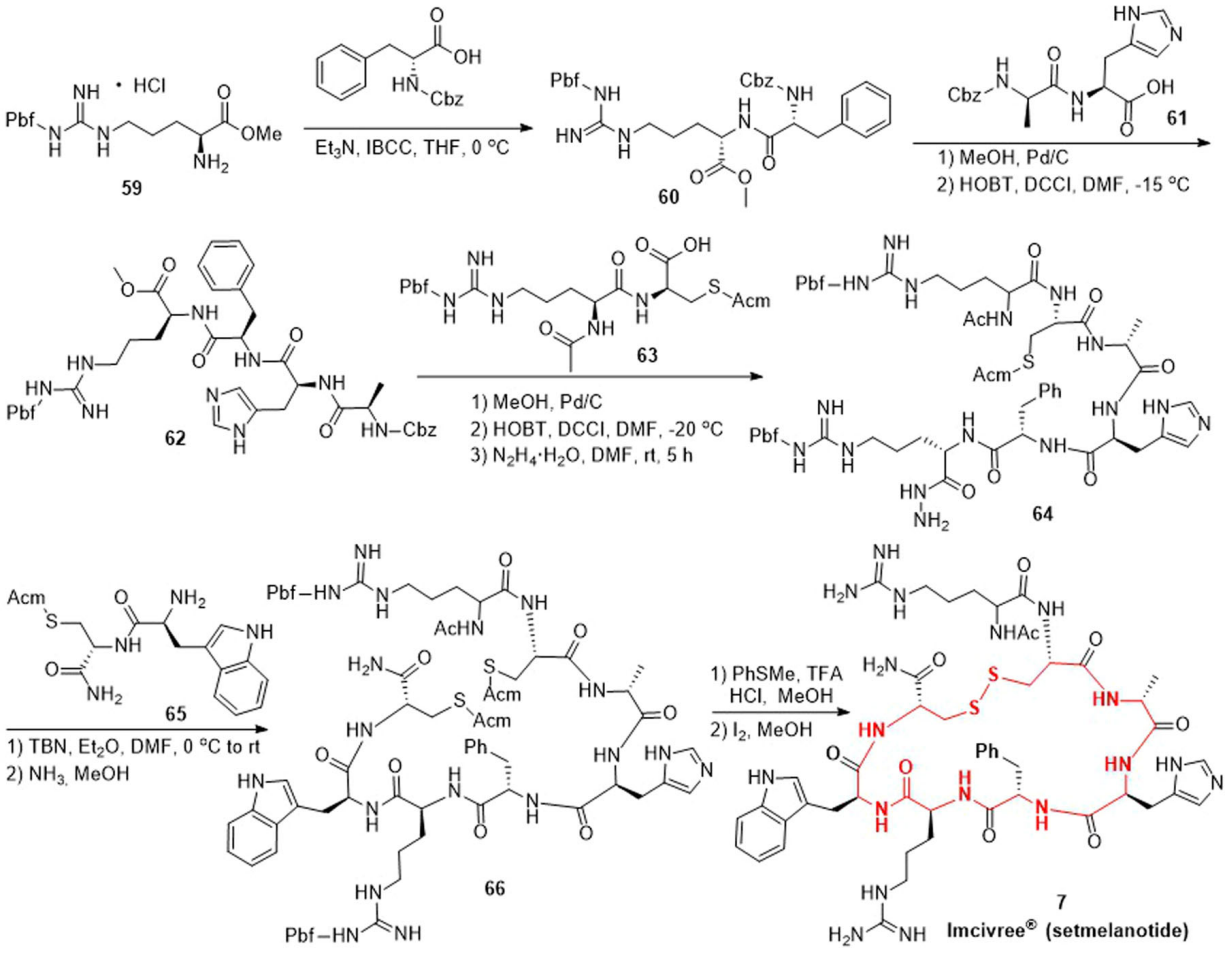
Synthesis of setmelanotide (**7**).

The synthesis of setmelanotide was presented in Scheme 7 using commercially available HCl·H‐Arg(Pbf)‐OMe as the starting material.^[^ ^77^ ^]^ Initially, condensation of HCl·H‐Arg(Pbf)‐OMe **59**, with Z‐d‐Phe‐OH in the presence of isobutyl chloroformate (IBCC) and triethylamine yielded Z‐d‐Phe‐Arg(Pbf)‐OMe **60**. Subsequently, the *N*‐Cbz protecting group of Z‐d‐Phe‐Arg(Pbf)‐OMe **60** was removed via Pd/C‐catalyzed hydrogenation. This intermediate then underwent condensation with Z‐d‐Ala‐His‐OH **61** in the presence of HOBt and dicyclohexylcarbodiimide (DCCI), resulting in the intermediate **62**. The intermediate **62** was hydrogenated under Pd/C conditions, followed by condensation with Ac‐Arg(Pbf)‐Cys(Acm)‐OH **63** in the presence of HOBt and DCCI. The resulting intermediate is then treated with hydrazine hydrate to generate the intermediate **64**. Compound **64** underwent a reaction with H‐Trp‐Cys(Acm)‐OMe **65** in the presence of *tert*‐butyl nitrite (TBN), followed by treatment with liquid ammonia to yield compound **66**. Compound **66** was then subjected to deprotection using TFA and hydrochloric acid (HCl). Subsequently, the formation of a disulfide bond was facilitated by iodine, ultimately leading to the production of setmelanotide (**7**). In 2023, The Anupam Bandyopadhyay research group reported a novel, highly efficient disulfide‐driven peptide macrocyclization method on solid support.^[^ ^78^ ^]^ This rapid (15‐min) process utilizes persulfate as a key additive in iodine‐mediated oxidative cyclization, eliminating the side products of classical iodine‐mediated peptide cyclization.^[^ ^77^ ^]^

### 3.3 Voclosporin (Lupkynis®)

Voclosporin (**8**, brand name Lupkynis®) is an oral calcineurin inhibitor immunosuppressant developed by Aurinia Pharmaceuticals, received its first approval in the United States in January 2021, for treating adults with active lupus nephritis (LN) in combination with background immunosuppressive therapy.^[^ ^79^, ^80^, ^81^ ^]^ LN, a severe complication of systemic lupus erythematosus (SLE), can cause irreversible kidney damage and contribute significantly to mortality. Despite existing treatments, there is an unmet need for effective and safer therapies due to slow response, high relapse rates, and toxicity concerns.^[^ ^79^, ^80^ ^]^ Voclosporin fills the void as the first oral therapy approved in the United States for this indication. It exhibits a dual mechanism of action in LN, including immunosuppression, achieved through inhibition of T‐cell activation, and antiproteinuria, achieved through stabilization of kidney podocytes.^[^ ^82^, ^83^, ^84^, ^85^ ^]^ Voclosporin (**8**) represents an innovative semisynthetic derivative of cyclosporin, featuring a unique molecular structure with 12 chiral centers and a 33‐membered macrocyclic ring structure. Initially, it was characterized as a hybrid entity, combining the terminal *E‐* and *Z*‐dienes of cyclosporin.^[^ ^86^, ^87^ ^]^ The macrocyclic ring structure of voclosporin is composed of a cyclic undecapeptide consisting of amino acids such as alanine, valine, and leucine.^[^ ^88^ ^]^ Additionally, it can exist in various distinct solid‐state forms, adding to its complexity and potential for diverse clinical applications. Besides LN, voclosporin's potential in treating dry eye and coronavirus disease 2019 (COVID‐19) in kidney transplant recipients is also being explored.^[^ ^89^, ^90^ ^]^

**Scheme 8:**
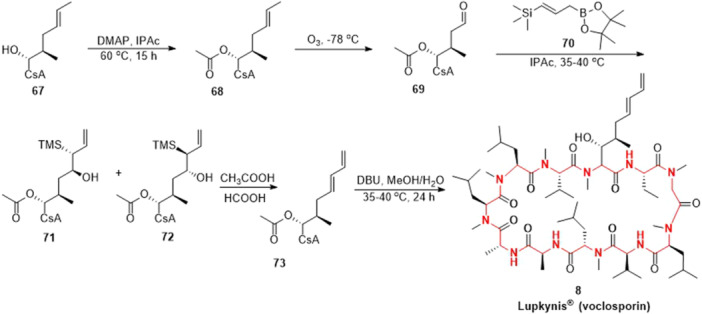
Synthesis of voclosporin (**8**).

There are several methods for the synthesis of voclosporin (**8**),^[^ ^91^, ^92^, ^93^ ^]^ in particular, Zhang and co‐authors reported an improved synthesis and crystallization method for voclosporin using cyclosporin A as the starting material (Scheme 8).^[^ ^90^ ^]^ The synthesis began with the preparation of the hydroxyl group of cyclosporin A **67** by using isopropyl acetate (IPAc) and DMAP as reagents. Subsequently, the intermediate **68** underwent ozonolysis to form acetyl cyclosporin A aldehyde **69**. Then, using (*E*)‐trimethyl(3‐(4,4,5,5‐tetramethyl‐1,3,2‐dioxaborolan‐2‐yl)prop‐1‐en‐1‐yl)silane (**70**) as an allyl reagent, *β*‐silanols with chemical structures of formula **71** and **72** were obtained. A Peterson elimination of the mixture of **71** and **72** under acetic acid and formic acid conditions yielded the intermediate **73** with an *E*/*Z* ratio of 98:2. Finally, 1,8‐diazabicyclo[5,4,0]‐7‐ene (DBU) was used as a hydrolyzing reagent to generate voclosporin (**8**) with an *E*/*Z* ratio of 97:3.

### 3.4 Terlipressin (Terlivaz®)

**Scheme 9:**
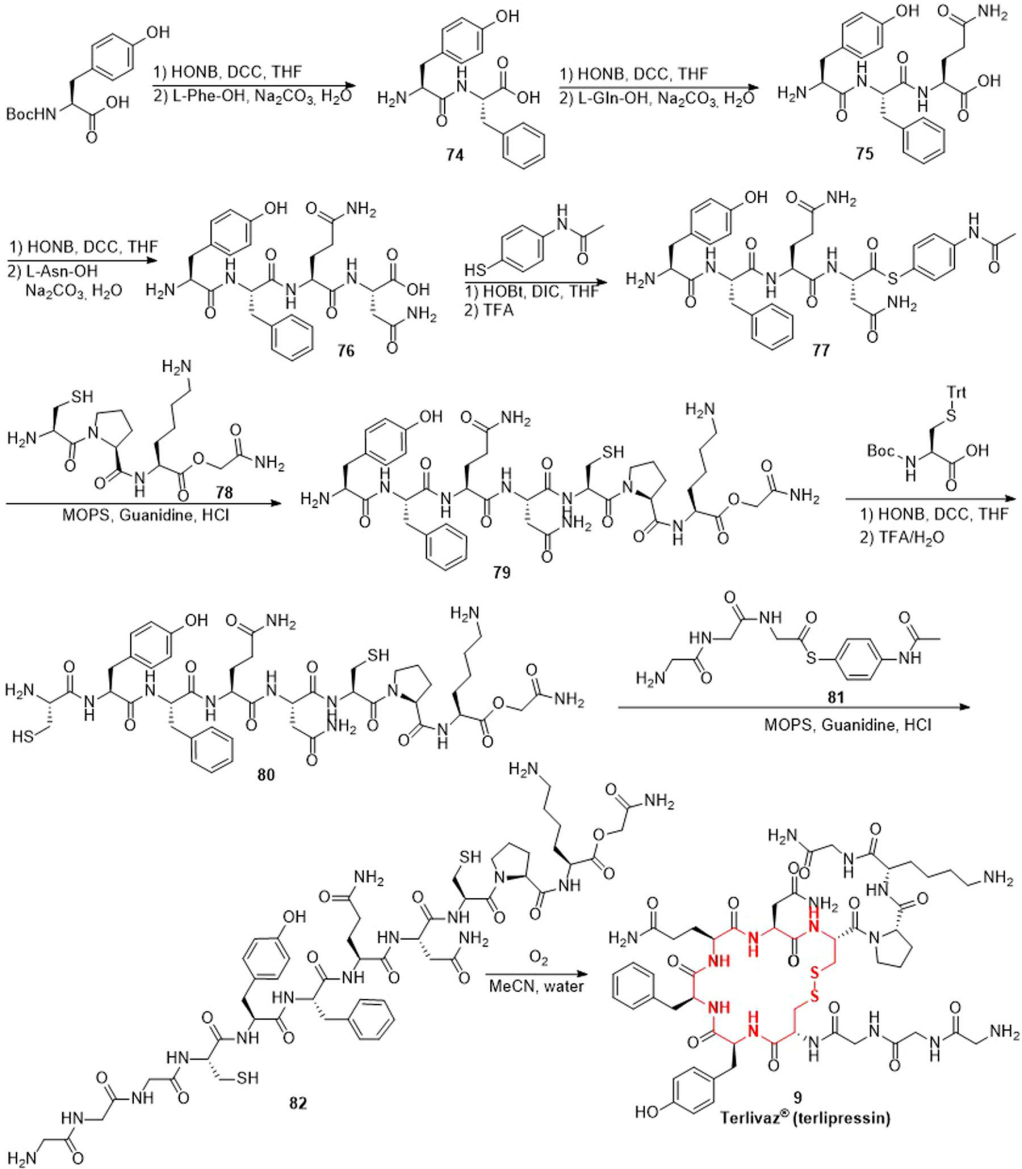
Synthesis of terlipressin **(9**).

Terlipressin (**9**, brand name Terlivaz®), is a vasopressin analog and potent vasoconstrictor, which was developed by Mallinckrodt Pharmaceuticals and received its FDA approval in September 2022.^[^ ^94^, ^95^ ^]^ This is the first drug approved by the FDA specifically for improving renal function in adult patients with hepatorenal syndrome (HRS). Terlipressin (**9**) achieves this by modulating the hemodynamic landscape, alleviating portal hypertension, and subsequently enhancing renal blood flow while boosting both effective arterial volume and pressure.^[^ ^94^, ^95^ ^]^ Structurally, terlipressin (**9**) is composed of nine amino acids, including l‐cysteine, glycine, lysine, l‐proline, and others, arranged in a unique conformation. Five of these amino acids form a 20‐membered macrocyclic peptide that is stabilized by a disulfide bridge, while the remaining four amino acids are attached from the *C*‐terminal of the cysteine residue. As a derivative of vasopressin, terlipressin undergoes several strategic modifications, leading to its ability to act as a potent vasopressin receptor agonist.^[^ ^96^, ^97^, ^98^, ^99^, ^100^ ^]^ One of the key advantages of terlipressin (**9**) over vasopressin lies in its extended half‐life and heightened selectivity for the V1 receptor.^[^ ^101^ ^]^ These favorable pharmacokinetic and pharmacodynamic properties bring practical benefits for patients, including ease of intravenous administration, and a reduced risk of rebound hypotension upon discontinuation.^[^ ^101^, ^102^ ^]^ Moreover, terlipressin's therapeutic potential extends beyond its primary indication. It is increasingly recognized as a promising candidate for the treatment of septic shock, a life‐threatening condition, and has also been proven to be a safe and effective therapeutic option for managing acute esophageal variceal bleeding.^[^ ^102^ ^]^ The preparation of terlipressin,^[^ ^78^, ^103^ ^]^ via a liquid‐phase segmented synthesis method was presented in Scheme 9.^[^ ^103^ ^]^ First, Boc‐Tyr(*t*Bu)‐OH underwent a coupling reaction with l‐Phe‐OH in the presence of *N*‐hydroxy‐5‐norbornene‐2,3‐dicarboxylic acid imide (HONB) and DCC, yielding the intermediate **74**. Subsequently, under similar conditions, compound **74** was coupled with l‐Gln‐OH and l‐Asn‐OH to give compound **76**. Compound **76** was first reacted with 4‐acetamidobenzenethiol in the presence of HOBt and DIC, and then deprotected with TFA to obtain the intermediate **77**. Using 3‐morpholinopropanesulfonic acid (MOPS) solution of guanidine hydrochloride as the buffer, compounds **77** and **78** were connected by the Native Chemical Ligation (NCL) method resulting in the intermediate **79**, which was coupled with Boc‐Cys(trt)‐OH in the presence of HONB and DCC, followed by deprotection using an aqueous solution of TFA. Then, compound **80** was connected with compound **81** affording the intermediate **82** after purification by RP‐HPLC (reverse‐phase high‐performance liquid chromatography). Finally, compound **82** was oxidized by O_2_ to give the target product terlipressin (**9**).

### 3.5 Rezafungin (Rezzayo®)

**Scheme 10:**
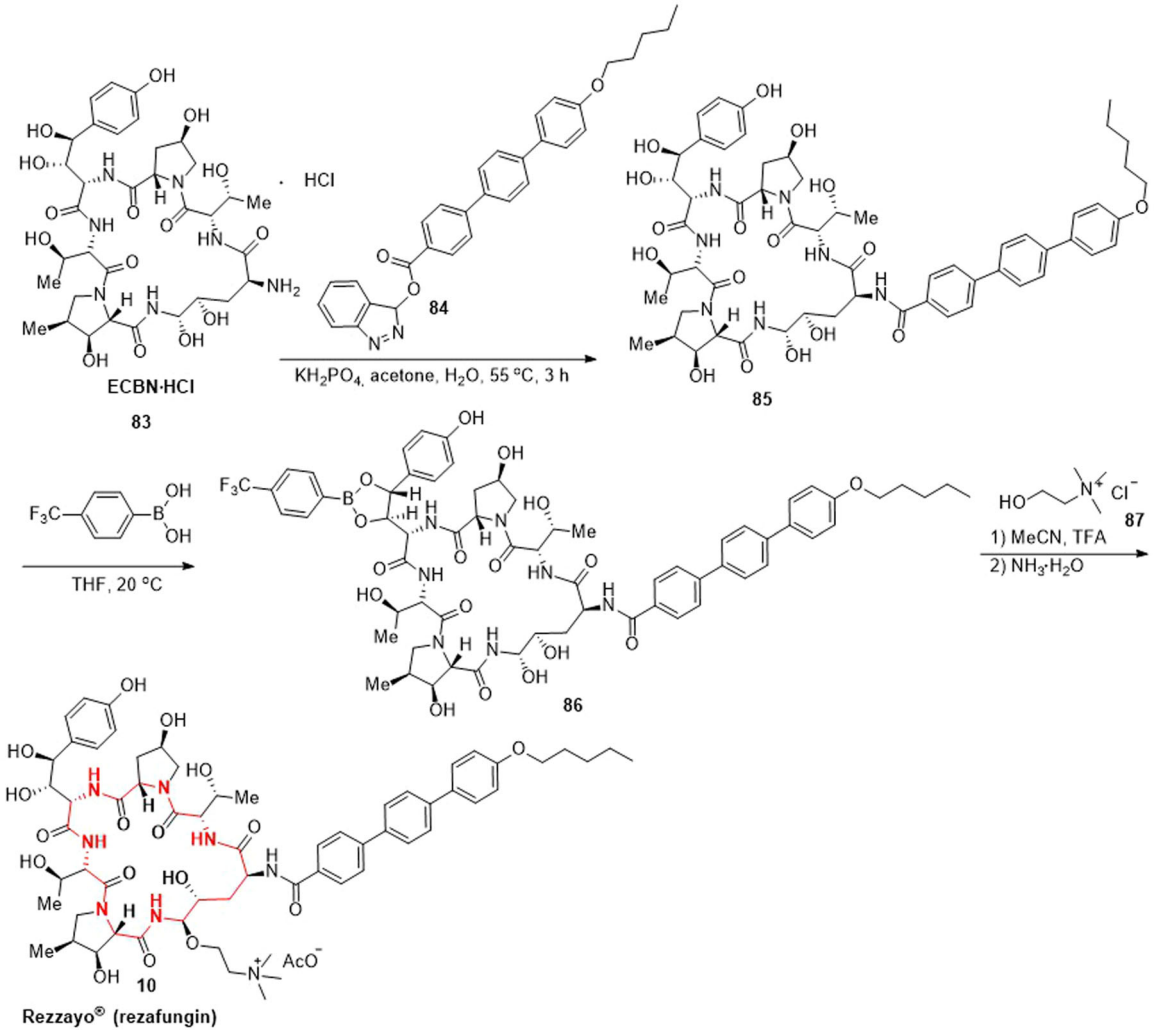
Synthesis of rezafungin (**10**).

Rezafungin (**10**, brand name Rezzayo®) is a second‐generation echinocandin antifungal agent and a novel β‐glucan synthase inhibitor developed by Cidara Therapeutics. In March 2023, rezafungin received approval from the FDA for the treatment of candidemia and invasive candidiasis in patients aged 18 and older who have limited therapeutic options or no alternative treatments available.^[^ ^104^ ^]^ Globally, over one billion people are affected by fungal infections, with approximately 11.5 million individuals suffering from severe infections, and more than 1.5 million deaths annually attributed to fungal diseases. The introduction of echinocandin drugs represents a significant breakthrough in the field of antifungal therapy.^[^ ^105^, ^106^, ^107^ ^]^ Rezafungin is a novel echinocandin antifungal agent that incorporates an alkoxytriphenyl side chain. When this side chain is removed through deacetylation, the cyclic core of rezafungin loses its antifungal activity, demonstrating the critical role of the alkoxytriphenyl side chain in maintaining the drug's activity.^[^ ^108^, ^109^, ^110^ ^]^ It is a structural analog of anidulafungin, sharing the same side chain and a very similar core 21‐membered macrocyclic hexapeptide structure with 14 chiral centers. Rezafungin (**10**) contains unnatural amino acids such as 4‐hydroxyproline, 3‐hydroxy‐4‐methylproline, dihydroxyornithine, and dihydroxyphenylalanine.^[^ ^110^ ^]^ However, the semiamine region of rezafungin is replaced by a choline amine ether, which reduces the chemical degradation that occurs in the semiamine region of anidulafungin, thereby enhancing the stability and solubility of rezafungin. Compared with first‐generation echinocandin drugs such as anidulafungin, caspofungin, and micafungin, rezafungin has been optimized in terms of chemical stability, tissue penetration, half‐life, and safety, aiming to achieve more effective therapeutic outcomes and a lower risk of side effects. Furthermore, rezafungin is undergoing further development with the intention of being used to prevent invasive fungal diseases in recipients of blood and bone marrow transplants.^[^ ^111^, ^112^ ^]^ The preparation of rezafungin (**10**) was shown in Scheme 10 with ECBN·HCl **83** as the starting material.^[^ ^113^, ^114^, ^115^ ^]^ ECBN·HCl **83** was obtained through the fermentation of *Aspergillus nidulans* and *Aspergillus* species, which was known as one of the natural cyclic hexapeptides with a linoleoyl side chain.^[^ ^116^, ^117^ ^]^ ECBN·HCl **83** reacted with alkoxytriphenyl **84** in the presence of potassium dihydrogen phosphate, resulting in the intermediate **85**. Subsequently, compound **85** underwent an esterification reaction with (4‐(trifluoromethyl)phenyl)boronic acid, yielding borate intermediate **86**. Finally, the intermediate **86** reacted with choline chloride **87** in the presence of TFA followed by deprotection by ammonium hydroxide to give the target rezafungin (**10**) (Scheme 10).

## 4 OTHER MACROCYCLIC DRUGS

### 4.1 Lorlatinib (Lorbrena®)

Lorlatinib (**11**, brand name Lorbrena®) is a reversible inhibitor of the anaplastic lymphoma kinase (ALK) and the kinase of the C‐Ros oncogene 1 (ROS1).^[^ ^118^ ^]^ After oral administration, the drug binds to both kinases, which results in an interruption of signaling pathways mediated by these kinases. The reason for this is that chromosomal rearrangements in the genes of ALK and ROS1 induce fusion kinases with constitutive activity.^[^ ^119^ ^]^ This in turn leads to the triggering of downstream signaling pathways that contribute to the development and progression of NSCLC.^[^ ^120^ ^]^ Lorlatinib is therefore used for the treatment of ALK‐positive NSCLC. It received approval for this indication in Japan in September 2018 and in the United States in November 2018.^[^ ^121^ ^]^ In March 2021, the approval was extended by the FDA to include first‐line treatment.^[^ ^122^, ^123^ ^]^

Lorlatinib (**11**) is a third‐generation inhibitor featuring the novelty that it has a macrocyclic structure in comparison to the acyclic representatives of the first and second generations. Lorlatinib was specifically developed to challenge ALK mutations to which first and second‐generation drugs are resistant.^[^ ^124^ ^]^ Another focus was on improving the penetration of the blood–brain barrier. Based on the chemical structure of crizotinib, structural modifications were carried out, for example, taking into account the lipophilic efficiency.^[^ ^125^ ^]^ This ultimately led to the amide (lactam) macrocycle structure of loratinib.

**Scheme 11:**
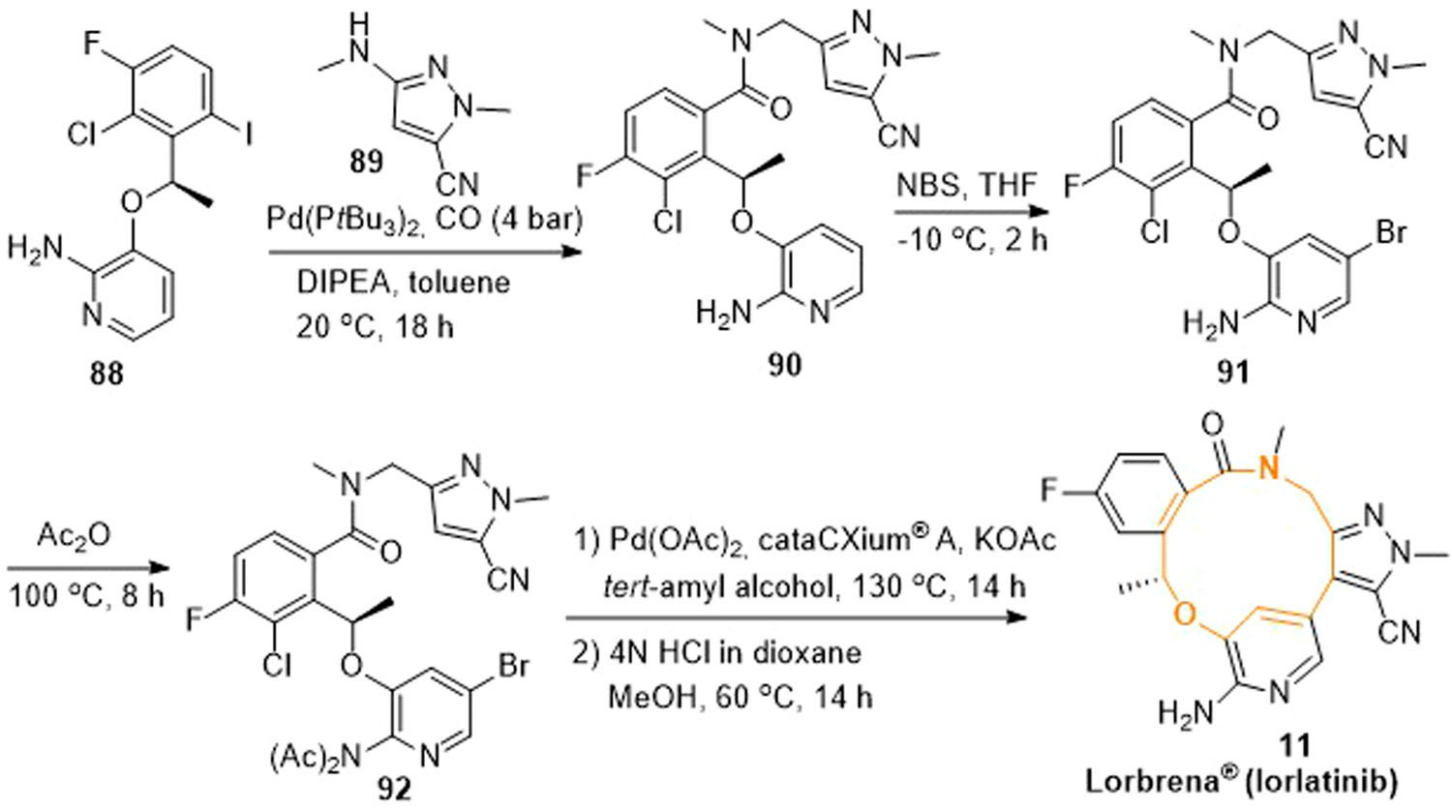
Synthesis of lorlatinib (**11**).

Lorlatinib (**11**) could be obtained according to the procedure presented in Scheme 11.^[^ ^126^, ^127^ ^]^ An essential step to synthesize the macrocyclic structure of loratinib was the approach featuring an intermolecular Suzuki coupling.^[^ ^125^ ^]^ Starting from (*R*)‐3‐(1‐(2‐chloro‐3‐fluoro‐6‐iodophenyl)ethoxy)pyridin‐2‐amine (**88**) and 2‐aminopyrazole derivative (**89**), the intermediate **90** was formed in the course of a Pd‐catalyzed amino‐carbonylation under carbon monoxide. The following bromination with a small excess (1.1 eq) of *N*‐bromo succinimide (NBS) yielded the intermediate **91**. A direct cyclization of the intermediate **91** led to unwanted by‐products and low yields of lorlatinib. Therefore, the aminopyridine structure was protected by acylation with acetic anhydride (Ac_2_O) to give the bis‐acetamide derivative **92**. Palladium‐catalyzed cross‐coupling led to intramolecular arylation and the formation of lorlatinib (**11**) as well as its singly and doubly protected derivatives. The latter two could also be converted to (**11**) by exhaustive deprotection in an acidic (hydrochloric acid) medium.

### 4.2 Moxidectin

Moxidectin (**12**) is a representative of the so‐called macrocyclic lactone parasiticides and serves as an anthelmintic modulator of glutamate‐gated chloride channels.^[^ ^128^ ^]^ The binding of moxidectin to the γ‐aminobutyric acid (GABA)‐mediated channel results in an influx of chloride ions and thus in enhanced permeability. This in turn leads to paralysis of the parasite and ultimately to its death.^[^ ^129^ ^]^ In June 2018, moxidectin was approved by the FDA for the treatment of human river blindness (onchocerciasis).^[^ ^130^ ^]^ Moxidectin is a semi‐synthetic derivative of nemadectin. The latter is a macrocyclic lactone produced by *Streptomyces cyaneogriseus subsp. Noncyanogenus*, hence belongs to the class of milbenmycins. It has antiparacitic properties, but only limited ectoparasitic activity. Based on this, the derivative moxidectin was further developed by the introduction of a methoxime moiety at C‐23.^[^ ^129^ ^]^ Moxidectin (**12**) also has broad spectrum potency against endo‐ and ectoparasites, thus belonging to the second generation of macrocyclic lactone parasiticides. Structurally, moxidectin is a 16‐membered, pentacyclic lactone fused to a spiroketal and a benzofuran core. Strikingly, it bears the unique methoxime feature.^[^ ^131^ ^]^

**Scheme 12:**
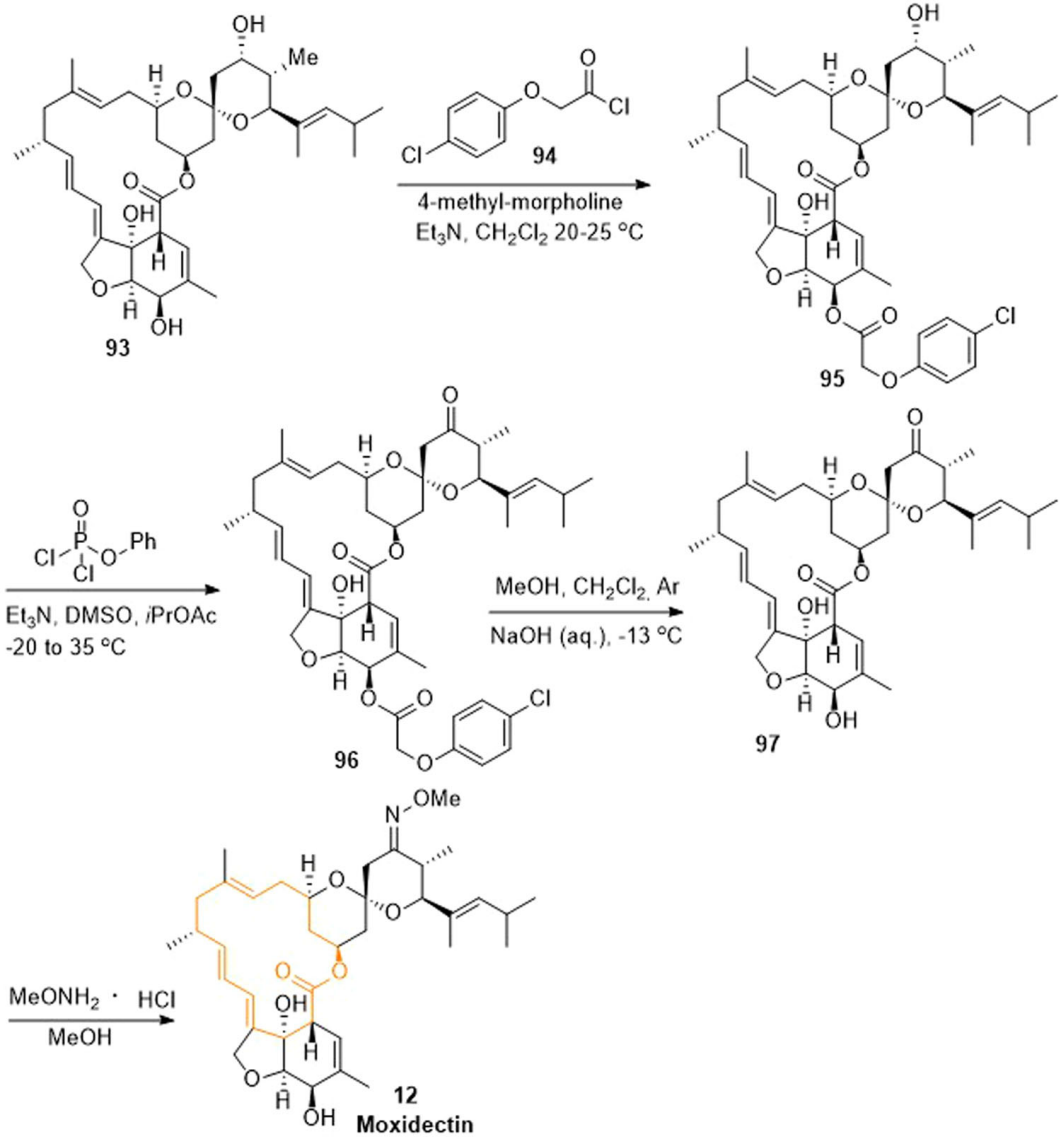
Semi‐chemical synthesis pathway of moxidectin (**12**) starting from nemadectin (**93**).

Moxidectin could be synthesized semi‐chemically from nemadectin (**93**) as shown in Scheme 12.^[^ ^130^, ^132^, ^133^ ^]^ First, the most active, secondary alcohol at C‐5 on the partially hydrogenated benzofuran core was protected by 2‐(4‐chlorophenoxy)acetyl chloride (**94**) to form the ester **95**. In the next step, the alcohol in position C‐23 of intermediate **95** was oxidized to ketone **96** via Swern oxidation, followed by deprotection of the alcohol at C‐5 again, resulting in the alcohol **97**. Final oximation of intermediate **97** with methoxylamine hydrochloride yielded moxidectin (**12**). The last step of the synthesis can be carried out with intermediate **97** dissolved in DCM and 1.5 equiv of methoxylamine hydrochloride or sodium acetate, respectively, under stirring for 10 h at 20–25°C.^[^ ^134^ ^]^ The deprotection step can also be carried out after the oxime formation step.^[^ ^135^ ^]^

Maulding and Kumar used 4‐nitrobenzoyl chloride for the purpose of protection, Corey's reagent (pyridinium chlorochromate, PCC) for the oxidation, and 4% alkali for the removal of the protecting group.^[^ ^136^ ^]^ Sapienza and co‐authors also employed 4‐nitrobenzoyl chloride, used 2‐iodoxybenzoic acid to oxidate, and alkaline hydrolysis for deprotection.^[^ ^137^ ^]^

### 4.3 Rifamycin (Aemcolo®)

Rifamycins are used therapeutically given their antibiotic effect exerted by inhibition of the bacterial DNA‐dependent RNA synthesis. In particular, rifamycins irreversibly bind to the β‐subunit of the DNA‐dependent RNA polymerase of prokaryotes. This hinders the binding of DNA to the polymerase. Thus, transcription cannot proceed and the protein biosynthesis is inhibited.^[^ ^138^ ^]^ In November 2018, an oral formulation of rifamycin SV (**13**, brand name Aemcolo®) received approval from the FDA for the treatment of travelers’ diarrhea induced by noninvasive strains of *Escherichia coli*.^[^ ^139^ ^]^ The use of the so‐called MultiMatrix® technology is a special feature here. This system facilitates the targeted release of the antibiotic drug in the lumen of the colon.^[^ ^140^, ^141^ ^]^ The therapeutic effect is then exerted locally there and side effects due to systematic absorption are reduced.^[^ ^139^ ^]^ Chemically, rifamycins belong to the ansamycin family of antibiotics. The structure of this class of antibiotics is reminiscent of a basket. An aromatic moiety (i.e., naphthalene core in the case of rifamycin SV **13**) is linked at two nonadjacent positions via a chain. The chain represents the handle (ansa in Latin) of the basket.^[^ ^138^ ^]^ This molecular architecture ultimately forms the macrocyclic structure of the drug.

**Scheme 13:**
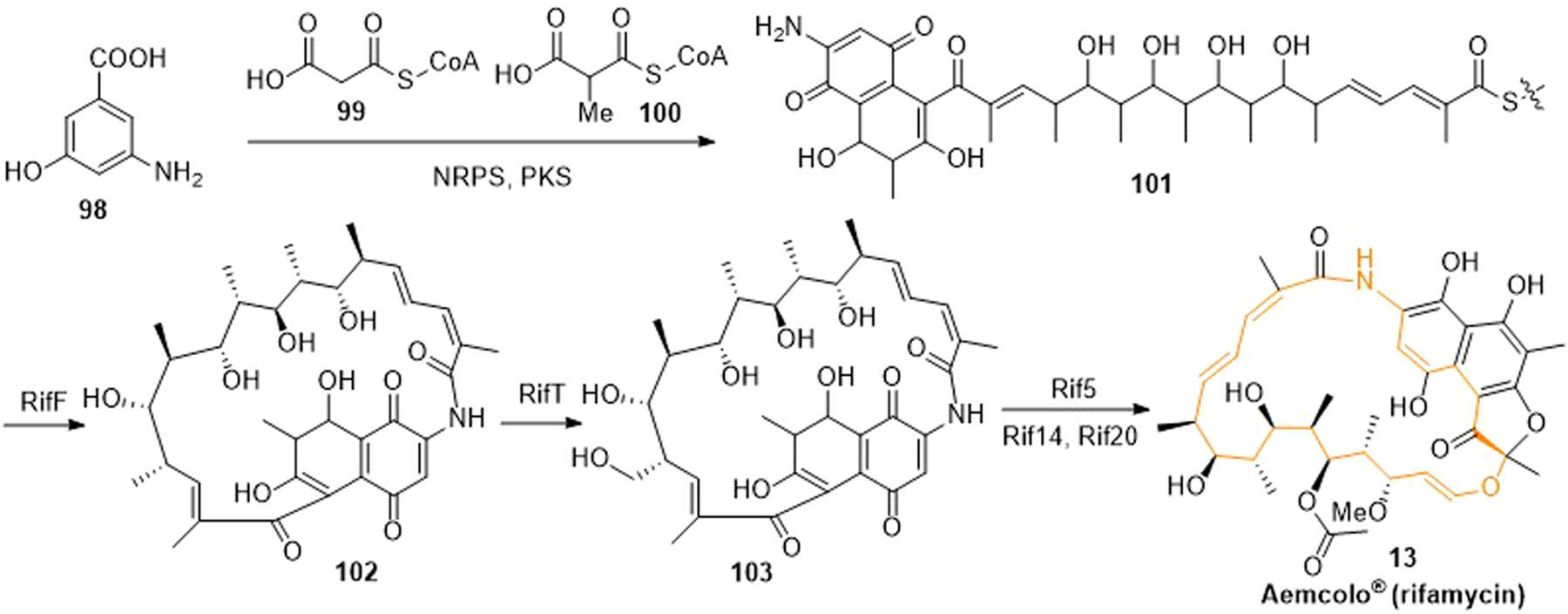
Biosynthetical route of rifamycin (**13**).

Rifamycin antibiotics were produced by fermentation of appropriate bacteria such as the actinobacterium *Amycolatopsis mediterranei*.^[^ ^142^ ^]^ An essential component of the biosynthesis was 3‐amino‐5‐hydroxybenzoic acid (AHBA, **98**) as the starting unit of the polyketide pathway. The synthetic route to the formation of AHBA was suggested in the literature.^[^ ^143^ ^]^ Based on AHBA, rifamycin SV (**13**) was obtained according to the following procedure (Scheme 13).^[^ ^144^ ^]^

AHBA was converted to the undecaketide (**101**) with two malonyl‐Coenzyme A (**99**) and eight methylmalonyl‐Coenzyme A (**100**) by a complex of a nonribosomal peptide synthase (NRPS) and a polyketide synthase (PKS). The PKS covered domains of acyltransferase (AT), acyl carrier protein (ACP), dehydratase (DH), ketoreductase (KR), and ketosynthase (KS).^[^ ^145^ ^]^ The formation of an intramolecular amide bond with the use of the protein RifF led to cyclization, generating proansamycin X (**102**). Further hydroxylation and dehydrogenation catalyzed by RifT yielded rifamycin W (**103**). The latter was converted to rifamycin SV (**13**) with the aid of the enzymes Rif5 (ketal formation), Rif14 (methylation of the hydroxyl group at C‐27), Rif20 (acetylation of the hydroxyl group at C‐25), and some unknown enzymes.

### 4.4 Lefamulin (Xenleta®)

Lefamulin (**14**, brand name Xenleta®) is a semi‐synthetic representative of the pleuromutilin class of antibiotics. In August 2019, it received approval from the FDA for treating community‐acquired bacterial pneumonia (CABP).^[^ ^146^ ^]^ The mechanism of action is distinctive and based on the inhibition of ribosomal protein synthesis in bacteria, which can reduce the risk of (cross) resistance to a large number of other antibiotic classes.^[^ ^147^ ^]^

In particular, the drug interacts with the catalytic center of the peptidyl transferase by binding to the A‐ and P‐site of the ribosomal subunit (50S) through hydrophobic interactions. As is typical for pleuromutilins, the tricyclic mutilin core of lefamulin (**14**) is oriented in a pocket near the A‐site. The side chain at C‐14 of the core is oriented toward the P‐site. This hinders the migration of the transfer RNA (tRNA) from the A‐site to the P‐site and thus the forming of a peptide binding.^[^ ^148^ ^]^ Inhibited translation within the protein biosynthesis leads to its disruption and ultimately to the lack of proteins that are necessary for bacterial growth.

The sidechain has a further significance. On the one hand, it increases the number of hydrogen bonds formed to the target, that is, the peptidyl transferase center. On the other, aspects such as increased solubility and metabolic stability are achieved. Therefore, lefamulin (**14**) can be applied by oral and intravenous formulations.^[^ ^149^ ^]^

**Scheme 14:**
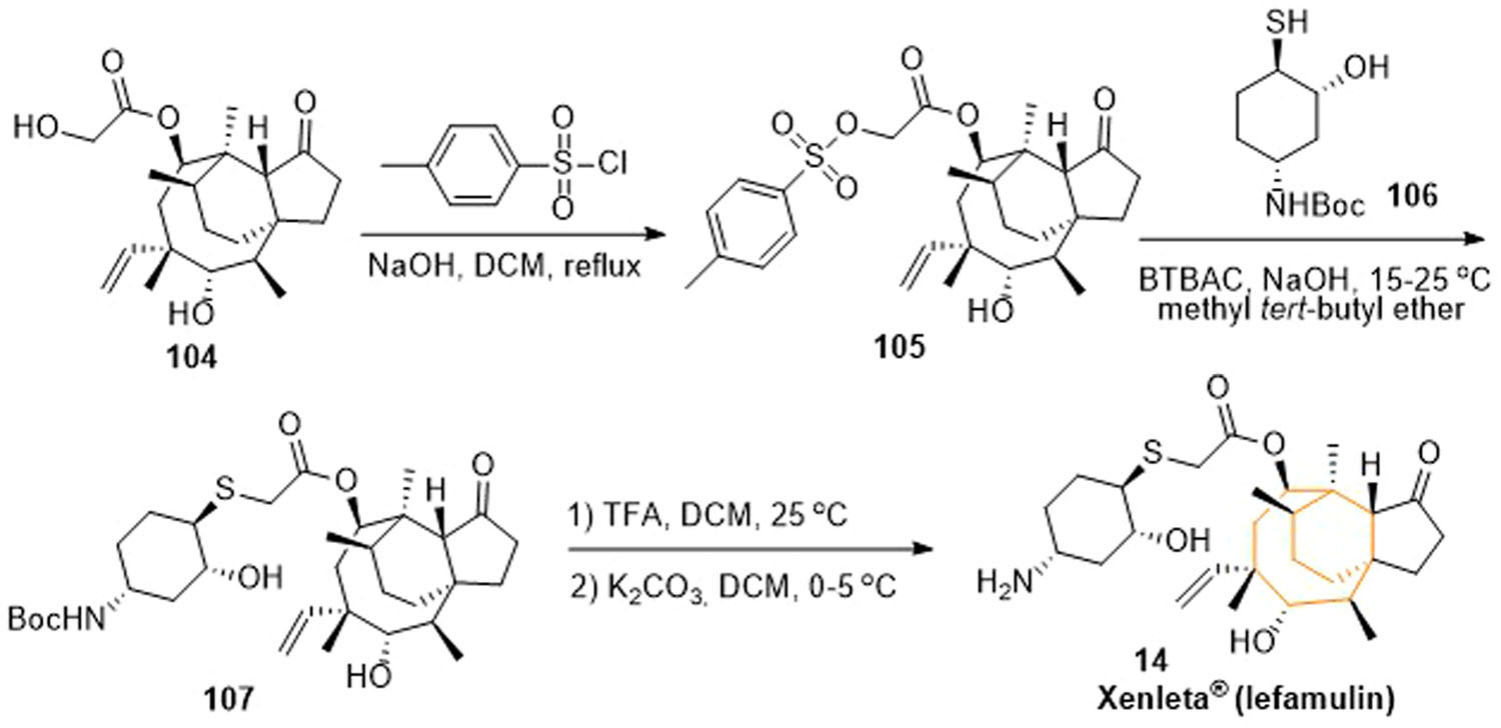
Synthesis of lefamulin (**14**).

Lefamulin could be synthesized from pleuromulin **104**, a natural compound bearing a medium‐sized ring. It could in turn be obtained biotechnologically through fermentation.^[^ ^150^ ^]^ A potential pathway to receive lefamulin was depicted in Scheme 14.^[^ ^151^ ^]^ The sulfonate **105** was obtained by conversion of the pleuromulin **104** with 4‐toluenesulfonyl chloride. Further reaction with the *N*‐protected cyclohexanethiol derivative **106** yielded the intermediate **107**. The carbamate moiety of **107** can finally be cleaved to give lefamulin (**14**) by exposing the amine via trifuloroacetic acid.

### 4.5 Pacritinib (Vonjo®)

Pacritinib (**15**, brand name Vonjo®) was first approved by the FDA in February 2022. It is a dual inhibitor of the Janus kinase 2 (JAK2) and the FMS‐like receptor tyrosin kinase 3 (FLT3). The drug is applied orally for the treatment of adults with low platelets (thrombocytopenia) who are affected by myelofibrosis.^[^ ^152^ ^]^ This is a type of bone marrow blood cancer that is due to the proliferation of myeloid stem cells.^[^ ^153^ ^]^ The latter can be caused by mutations in the Philadelphia chromosome and in the Janus kinase.^[^ ^154^ ^]^ Representatives of the JAK family are involved in the regulation of hematopoiesis. As a result, the development of inhibitors of tyrosine kinases of the JAK family has emerged as a promising therapeutic approach in recent years, including myelofibrosis.^[^ ^155^ ^]^ The small molecule pacritinib is selective for the isoform JAK2 as it does not inhibit JAK1 or JAK3.^[^ ^156^ ^]^ It is dysfunction in the JAK2 signaling pathway that is associated with myelofibrosis. Compared with the previously approved JAK inhibitors, pacritinib (**15**) is a macrocyclic compound. Structural design led to improved water solubility given by the pyrrolidine group.^[^ ^157^ ^]^

**Scheme 15:**
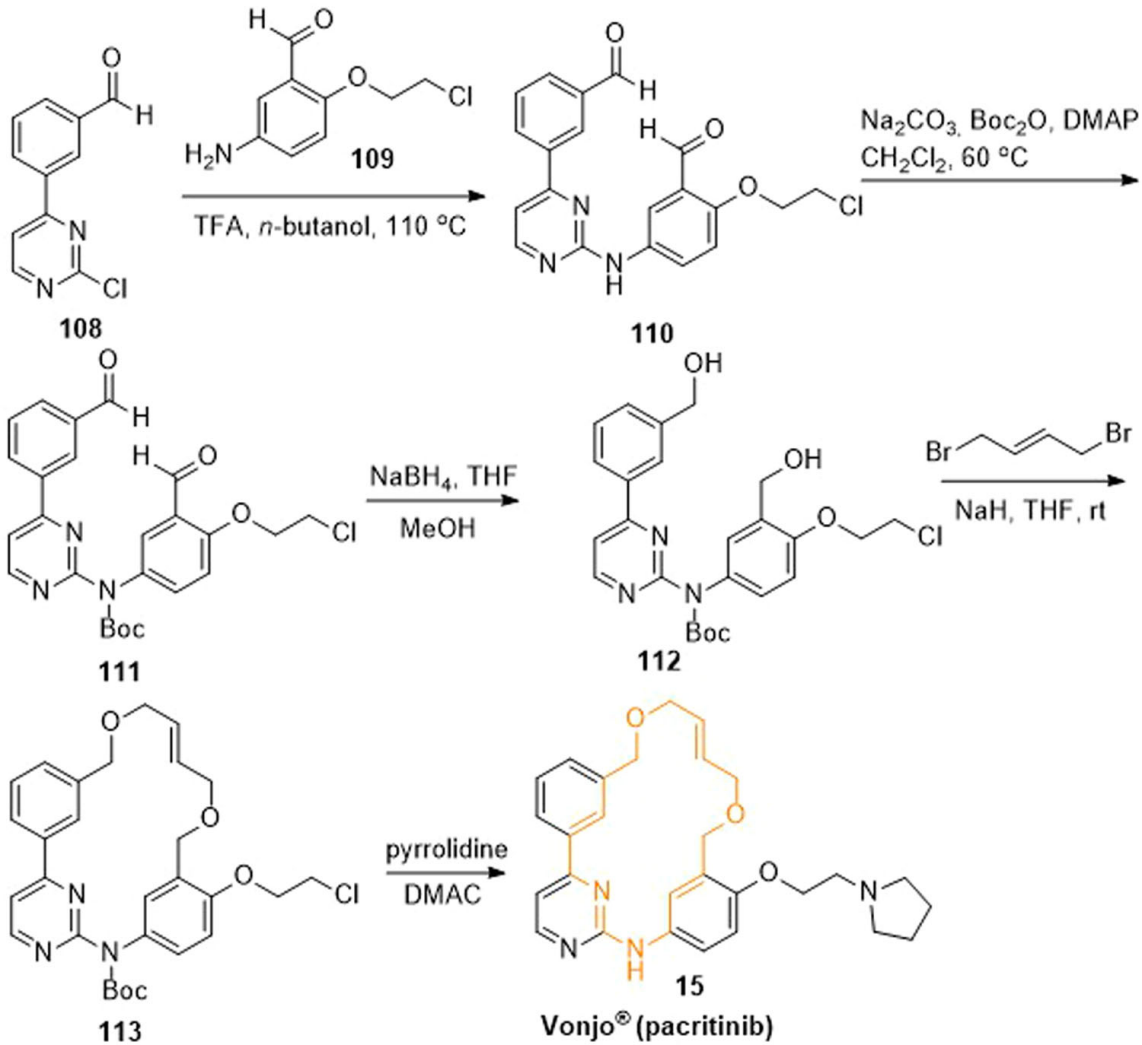
Synthesis of pacritinib (**15**).

The synthesis of pacritinib could be carried out in five steps as shown in Scheme 15.^[^ ^152^ ^]^ First, 3‐(2‐chloropyrimidin‐4‐yl)benzaldehyde (**108**) and 5‐amino‐2‐(2‐chloroethoxy)benzaldehyde (**109**) underwent a substitution reaction to give the intermediate **110**. Next, the secondary amine of the intermediate **110** was *N*‐protected using di‐*tert*‐butyldicarbonate resulting in the intermediate **111**. Both benzaldehyde moieties of the intermediate **111** were then reduced to benzyl alcohols mediated by sodium borohydride. The fourth step of the synthesis sequence comprised cyclization. For this purpose, alcohol **112** was reacted with the bis‐functionalized (*E*)‐1,4‐dibromobut‐2‐ene under basic conditions as part of a William ether synthesis yielding the cyclic intermediate **113**. Finally, pacritinib (**15**) was obtained following a nucleophilic substitution reaction with pyrrolidine. Further, very similar approaches to the synthesis of pacritinib can be found in the literature.^[^ ^157^, ^158^ ^]^

### 4.6 Vonoprazan, amoxicillin, clarithromycin (Voquezna Triple Pak®)

In May 2022, the FDA approved the preparation Voquezna triple pak consisting of the active ingredients vonoprazon, amoxicillin, and clarithromycin. The approval was for the treatment of infections in adults caused by *Helicobacter pylori*. In October 2023, this pharmaceutical was finally also registered for the therapy of erosive esophagitis.^[^ ^159^ ^]^ Vonoprozan inhibits the secretion of gastic acid. It targets the proton pump enzyme H^+^/K^+^‐ATPase and acts as a potassium‐competitive acid blocker.^[^ ^159^, ^160^ ^]^ Amoxicillin is commonly combined with proton pump inhibitors for the treatment of *Helicobacter pylori* infections.^[^ ^161^ ^]^ It is a representative of the β‐lactam antibiotics, in particular of the aminopenicillins, and is one of the most commonly used penicillins.^[^ ^162^ ^]^ As a target, penicillins interfere in the cell wall synthesis of bacteria by preventing the linking of new peptidoglycans. As a result, a bactericidal effect is exerted.

However, the active ingredient clarithromycin (**16**) within the triple combination of Voquezna is actually much more interesting. Clarithromycin is a macrolide antibiotic, that is, a macrocyclic compound comprising a lactone moiety. It inhibits bacterial protein synthesis. After reversible binding to the 50S subunit of the ribosomes, it prevents the transfer of amino acids to elongate the peptide chain (translocation).^[^ ^163^ ^]^ Clarithromycin (**16**) is commonly used for *Helicobacter pylori* infections.^[^ ^164^ ^]^

Chemically, two sugars (desosamine and cladinose) are bound to the 14‐membered macrolide ring in the structure of clarithromycin (**16**).^[^ ^165^ ^]^ Clarithromycin is also known as 6‐*O*‐methylerythromycin and thus represents a structural further development of erythromycin A. The additional methylation of the hydroxyl group at C‐6 (leading to a methoxy group) prevents the intramolecular formation of the erythromycin‐6,9‐hemiketal. The latter is formed by the reaction of the C‐6 hydroxyl group with the C‐9 carbonyl. Hence, methylation increases the stability of clarithromycin compared to erythromycin,^[^ ^166^ ^]^ especially against gastric acid.^[^ ^167^ ^]^

**Scheme 16:**
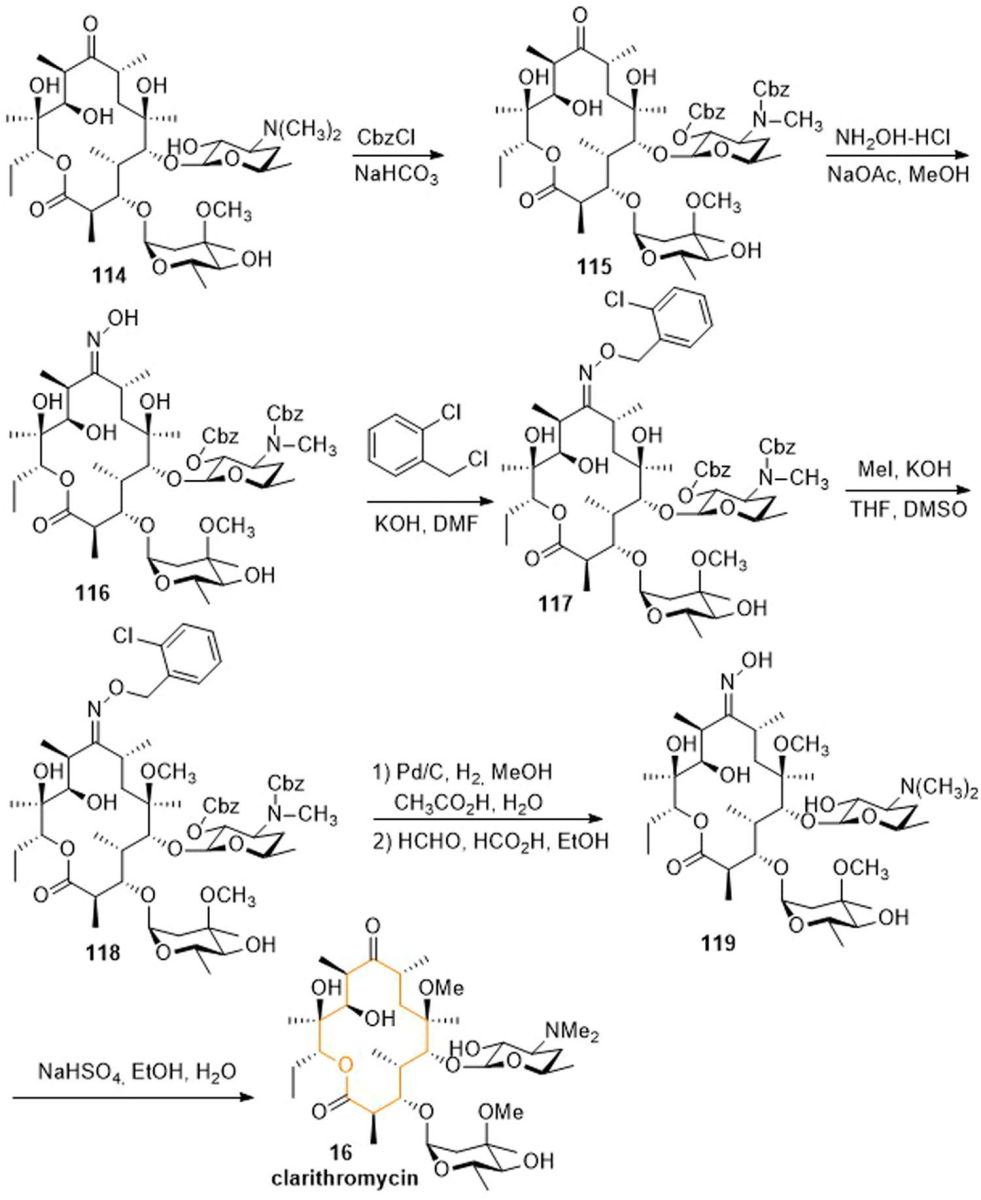
Synthesis of clarithromycin (**16**).

Clarithromycin (**16**) was produced semi‐synthetically from the macrolide antibiotic erythromycin A and represents the 6‐*O*‐methylerythromycin. In the synthesis of clarithromycin starting from erythromycin A, methylation must therefore take place at C‐6, which was shown in Scheme 16.^[^ ^168^ ^]^ First, erythromycin A **114** was converted to the *O,N*‐dicarbobezoxydes‐*N*‐methylerythromycin **115** using benzyl chlorocarbonate to protect the hydroxyl and amino groups of the sugar residue desosamine. In the next step, the C‐9 carbonyl moiety was converted to oxime **116** using hydroxylamine, followed by protection with 2‐chlorobenzyl chloride to give the bis‐carbobenzoxy 9‐oxime derivative **117**. Methylation with methyl iodide yielded the 6‐*O*‐methyl congener **118**. The carbobenzoxy groups (Cbz) were removed by hydrogenation and the *N*‐methylation was carried out by an Eschweiler–Clarke reaction, which led to 6‐*O*‐methylerythromycin‐9‐oxime **119**. Deoximation was accomplished by reaction with sodium hydrogen sulfite to form clarithromycin (**16**).

### 4.7 Repotrectinib (Augtyro®)

Repotrectinib (**17**, brand name Augtyro®) was approved in the United States in November 2023. It is used in an orally bioavailable form for the treatment of locally advanced or metastatic NSCLC that is positive for the proto‐oncogene tyrosine protein kinase ROS1.^[^ ^169^ ^]^ Repotrectinib (**17**) serves as an inhibitor of ROS1 and of the tropomyosin receptor tyrosine kinases A, B, and C (TRKA, TRKB, and TRKC).^[^ ^170^ ^]^ Since repotrectinib can circumvent the challenge of acquired resistance of first‐generation ROS1 inhibitors (i.e., crizotinib and entrectinib), it is considered a representative of the next‐generation of ROS1 and TRK inhibitors.^[^ ^171^ ^]^ Structurally, repotrectinib (**17**) is a small molecule with a macrocyclic lactam core. However, the molecule is rather condensed and has a small surface area for binding to the tyrosine kinases. As a result, repotrectinib is less affected by steric hindrance as a consequence of mutations (which ultimately lead to resistance to first‐generation drugs). Therefore, repotrectinib (**17**) is also effective against mutated fusions of ROS1.^[^ ^172^ ^]^ Repotrectinib has one single fluorination of a phenyl core in 4‐position to the ether moiety. This counteracts the oxidative metabolic decomposition of the drug.^[^ ^173^ ^]^

**Scheme 17:**
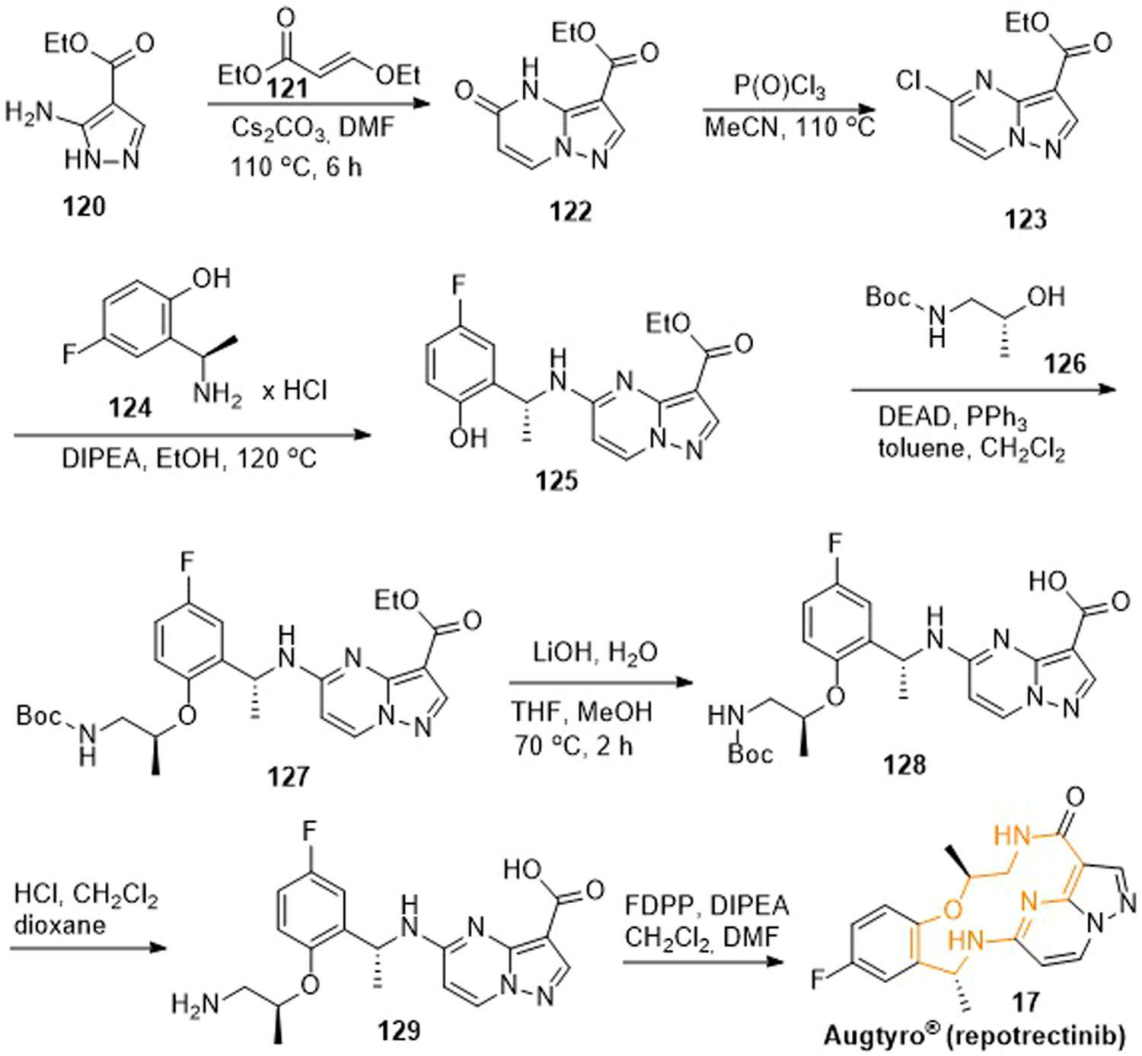
Synthesis of repotrectinib (**17**).

The synthetic approach to repotrectinib covered several steps, starting from ethyl 5‐aminopyrazole‐4‐carboxylate (**120**) (Scheme 17).^[^ ^173^, ^174^ ^]^ This precursor **120** cyclized with ethyl 3‐ethoxyacrylate (**121**) to ethyl 5‐oxo‐4,5‐dihydropyrazolo[1,5‐*a*]pyrimidine‐3‐carboxylate (**122**). Chlorination with phosphoryl chloride gave ethyl 5‐chloropyrazolo[1,5‐*a*]pyrimidine‐3‐carboxylate (**123**). The pyrimidine **123** was coupled with 2‐[1(*R*)‐aminoethyl]‐4‐fluorophenol hydrochloride (**124**) within a nucleophilic substitution to afford the secondary amine **125**. The phenolic moiety of intermediate **125** was converted to the alkyl aryl ether **127** using *N*‐Boc‐1‐amino‐2‐(*R*)‐propanol (**126**) according to a Mitsunobu reaction. Then, the ethyl ester was alkaline‐cleaved with lithium hydroxide resulting in the carboxylic acid **128**, followed by deprotection of the Boc‐groups in hydrochloric acid to yield the primary amine **129**. An intramolecular amide formation employing diphenylphosphinic acid pentafluorophenyl ester (FDPP) led to cyclization and provided the desired repotrectinib (**17**).

## 5 CONCLUSIONS AND OUTLOOK

Functionalized macrocycles and medium‐sized rings have received considerable attention in recent years, and are widely applied in many scientific fields, in particular medicinal chemistry due to their unique structure. Seventeen examples are presented, which include five macrocyclic radiopharmaceuticals, five cyclic peptide drugs, and seven other macrocyclic drugs. These drugs represent such therapeutic areas as treating cancer, chronic weight management, active lupus nephritis, hepatorenal syndrome, candidemia and invasive candidiasis, onchocerciasis, myelofibrosis and thrombocytopenia, and more. A wide range of ring sizes (from tens to thirties membered) is observed, and the largest macrocycle contains even 33 atoms. Also, bridged cycle and spirocycle skeletons occur in two drugs lurbinectedin (Zepzelca®) (**6**) and lefamulin (Xenleta®) (**14**).

It can be noticed that N, O, and S are the most appearing heteroatoms in these drugs. Also, a relative proportion (about 60%) of molecules is bearing amino acids and peptides. In particular, five macrocyclic radiopharmaceuticals all feature cyclic peptide units. Another important trend observed in these drugs is that almost all of these compounds are chiral possessing up to fifteen stereogenic carbons, and only one drug, pacritinib (Vonjo®) (**15**), is an achiral compound.

In summary, the question arises as to what can be learned from the 17 representative drugs of the three subgroups, that is, macrocyclic radiopharmaceuticals, cyclic peptides, and the group of other compounds focused on in this review.

All the five radiopharmaceuticals discussed herein carry a derivative of the DOTA group. This core comprises a 12‐membered tetraaza ring which, as a chelator, has a high affinity for divalent and trivalent cations.^[^ ^175^ ^]^ This makes DOTA suitable for the complexation of radioactive metal ions such as ^177^Lu^3+^, ^64^Cu^2+^, and ^68^Ga^3+^, as well as strongly paramagnetic cations like Gd^3+^ (in the chemical structure of drugs **1** and **3**, **2**, **4**, and **5**, respectively). The DOTA scaffold or cyclen derivatives in general are thus introduced into the chemical structure as a means for the purpose of complexation. It can be introduced through a DOTA(*t*Bu)_3_ coupling, whereby the *tert*‐butyl ester can subsequently be cleaved again in an acidic‐mediated manner, for example, using TFA.

In the case of the peptide‐based macrocyclic drugs, the bifunctionality of amino acids representing the subunits of peptides can be exploited. By nucleophilic attack of the amino group on the carbonyl carbon atom of the carboxylic group of another amino acid, a chain can initially be built by the formation of a peptide bond. Such an extended chain can then cyclize through intramolecular peptide bonding. Due to the variety of amino acids given their side groups, diverse molecules are also conceivable. Special attention is paid to the stereochemical diversity as amino acids belong to the chiral pool. Among the amino acids, cysteine with its thiol group plays a special role. This is because the formation of a disulfide bridge opens up a further possibility for cyclization. This can take place, for example, starting from acetamidomethyl (Acm)‐protected thiols (e.g., in the synthesis of setmelanotide **7**) or by oxidation of free thiols (e.g., in the synthesis of terlipressin **9**). Consequently, the incorporation of thiols into the amino acid backbone paves the way to form medium‐sized rings or macrocyclic therapeutics through disulfide bridges.^[^ ^176^ ^]^

For the third subset of compounds addressed in this review, the possibilities seem even more manifold. In principle, the formation of cyclic compounds is based on a general pattern for all representatives. The precursors of the cyclic molecules have two functional groups that can react with each other. This bifunctionality can be used for cyclization by intramolecular reaction. Similar to peptide‐based drugs, the ring closure can be achieved by forming an intramolecular peptide bond (amine reacts with carbonyl in the example of rifamycin **13** and repotrectinib **17**). A bis‐functional alkyl halide can be used to link two alcohols by means of an ether bridge (e.g., in the synthesis of pacritinib **15**). Cross‐coupling can also be exploited for intramolecular arylation (e.g., in the synthesis of lorlatinib **11**). Finally, there are also examples where the synthesis of already cyclic reactants can be assumed (e.g., moxidectin **12**, lefamulin **14**, clarithromycin **16**). Overall, however, it is noticeable that macrocycles appear to be preferred to medium‐sized rings due to the transannular strains that would be formed in the smaller compounds.

Nowadays, the incorporation of macrocycles and medium‐sized rings is embedded in activities in medicinal chemistry, resulting in a major impact on contemporary organic chemistry. One can expect a continuous increase in the number of macrocycle‐containing drugs on the pharmaceutical market in the near future.

## CONFLICTS OF INTEREST STATEMENT

The authors declare no conflicts of interest.

## Data Availability

Data sharing is not applicable to this article as no datasets were generated or analyzed during the current study.
